# ZEB1 confers chemotherapeutic resistance to breast cancer by activating ATM

**DOI:** 10.1038/s41419-017-0087-3

**Published:** 2018-01-19

**Authors:** Xiang Zhang, Zhen Zhang, Qing Zhang, Quansheng Zhang, Peiqing Sun, Rong Xiang, Guosheng Ren, Shuang Yang

**Affiliations:** 1 Department of Endocrine and Breast Surgery, The First Affiliated Hospital of Chongqing Medical University, Chongqing, 400016 China; 2 Tianjin Key Laboratory of Tumor Microenvironment and Neurovascular Regulation, Medical College of Nankai University, Tianjin, 300071 China; 3 Tianjin Key Laboratory of Organ Transplantation, Tianjin First Center Hospital, Tianjin, 300192 China; 4 Department of Cancer Biology, Wake Forest University School of Medicine, Winston-Salem, NC 27157 USA

## Abstract

Although zinc finger E-box binding homeobox 1 (ZEB1) has been identified as a key factor in the regulation of breast cancer differentiation and metastasis, its potential role in modulating tumor chemoresistance has not been fully understood. Here, through the study of specimens from a large cohort of human breast cancer subjects, we showed that patients with tumors that expressed high levels of ZEB1 responded poorly to chemotherapy. Moreover, ZEB1 expression was positively correlated with expression of B-cell lymphoma-extra large (Bcl-xL) and cyclin D1, which are key components of tumor chemoresistant mechanisms. At the molecular level, ectopic expression of ZEB1 impaired the responsiveness of breast cancer cells to genotoxic drug treatment, such as epirubicin (EPI). During this process, ZEB1 transcriptionally activated the expression of ataxia-telangiectasia mutated (ATM) kinase by forming a ZEB1/p300/PCAF complex on its promoter, leading to increased homologous recombination (HR)-mediated DNA damage repair and the clearance of DNA breaks. Using a nude mouse xenograft model, we further confirmed that ectopic expression of ZEB1 decreased breast cancer responsiveness to EPI treatment in vivo. Collectively, our findings suggest that ZEB1 is a crucial determinant of chemotherapeutic resistance in breast cancer.

## Introduction

Breast cancer is the most common malignancy among women worldwide and one of the leading causes of cancer death^1,2^. For breast cancer treatment, genotoxic chemotherapy using drugs such as anti-metabolites, topoisomerase inhibitors and anthracyclines, is a principal approach, which destroys cancer cells by inducing irreparable DNA damage. These therapeutic agents are particularly important for the treatment of breast cancer that are not suitable for or refractory to endocrine therapies. However, a major cause of failure in genotoxic drug treatment is intrinsic and therapy-induced chemoresistance due to enhanced DNA repair in tumor cells^3,4^. To overcome this problem, it is necessary to elucidate the mechanisms of resistance to chemotherapy and develop new chemosensitizers.

The primary mechanism of action of genotoxic drugs is to interfere with enzymes involved in DNA replication. These drugs can also induce DNA intercalation and damage, which ultimately results in DNA lesions in the forms of double-stranded breaks (DSBs)^5^. The cellular response to DNA damage, known as the DNA damage response (DDR), involves the recognition of DNA damage, the activation of cell cycle checkpoints, transcription programs, DNA damage repair, and apoptosis, if the damage is irreparable^6^. DSBs are predominantly repaired by two mechanisms, homologous recombination (HR) and non-homologous end joining^7,8^. Ataxia-telangiectasia mutated (ATM) kinase, a keystone in controlling genomic stability, plays a critical role in DDR and HR repair through a mechanism that is currently not well understood^9,10^. Nevertheless, it has been proposed that upon DSBs, ATM kinase is activated in a process involving autophosphorylation on serine (Ser^1981^) and in turn hyperphosphorylates downstream effecters, such as H2AX, CHK2 and p53 binding protein 1 (53BP1), ultimately leading to the recruitment of DNA repair proteins to the sites of damage^11–15^.

Zinc finger E-box binding homeobox 1 (ZEB1) is a transcription factor that modulates cell differentiation and tissue-specific functions^16–18^. ZEB1 expression is implicated in the differentiation of multiple cell lineages, including bone-^17,19^, smooth muscle^20^, neural-^21^, and T cells^22^. Recent studies further demonstrate that ZEB1 acts as a driver of epithelial to mesenchymal transition (EMT) and cancer progression, due to its pivotal role in the downregulation of epithelial genes, such as E-cadherin and the miR-200 family of microRNAs^23–26^. Aberrant expression of ZEB1 has been observed in many types of human cancers, including uterine cancer^27^, pancreatic cancer^28^, osteosarcoma^29^, lung cancer^30^, liver cancer^31^, gastric cancer^32^, colon cancer^33^, and breast cancer^34^. Additionally, ZEB1 is linked to a chemoresistant phenotype in cancer cells^35,36^. For instance, silencing ZEB1 expression reduces both invasion and the resistance to temozolomide, which is a standard chemotherapeutic drug for glioblastoma^35^. In pancreatic cells, the EMT status and expression level of ZEB1 correlate with the resistance to chemotherapeutic agents including gemcitabine, 5-fluorouracil (5-FU), and cisplatin^36^. However, the molecular mechanisms by which ZEB1 mediates chemoresistance are yet to be determined.

In this study, we provide evidence that the EMT regulator ZEB1 plays an important role in breast cancer chemoresistance by increasing HR-mediated DNA damage repair. During this process, ZEB1 induces ATM expression by forming a ZEB1/p300/PCAF complex on the ATM promoter. Notably, we demonstrated that patients with tumors that highly express ZEB1 have a dramatically weaker response to genotoxic drug-based chemotherapy. Our data collectively have identified a molecular mechanism underlying ZEB1-mediated chemoresistance, indicating that ZEB1 may be a potential target for breast cancer treatment.

## Results

### Elevated ZEB1 expression correlates with chemoresistance in human breast cancer

**Fig. 1:**
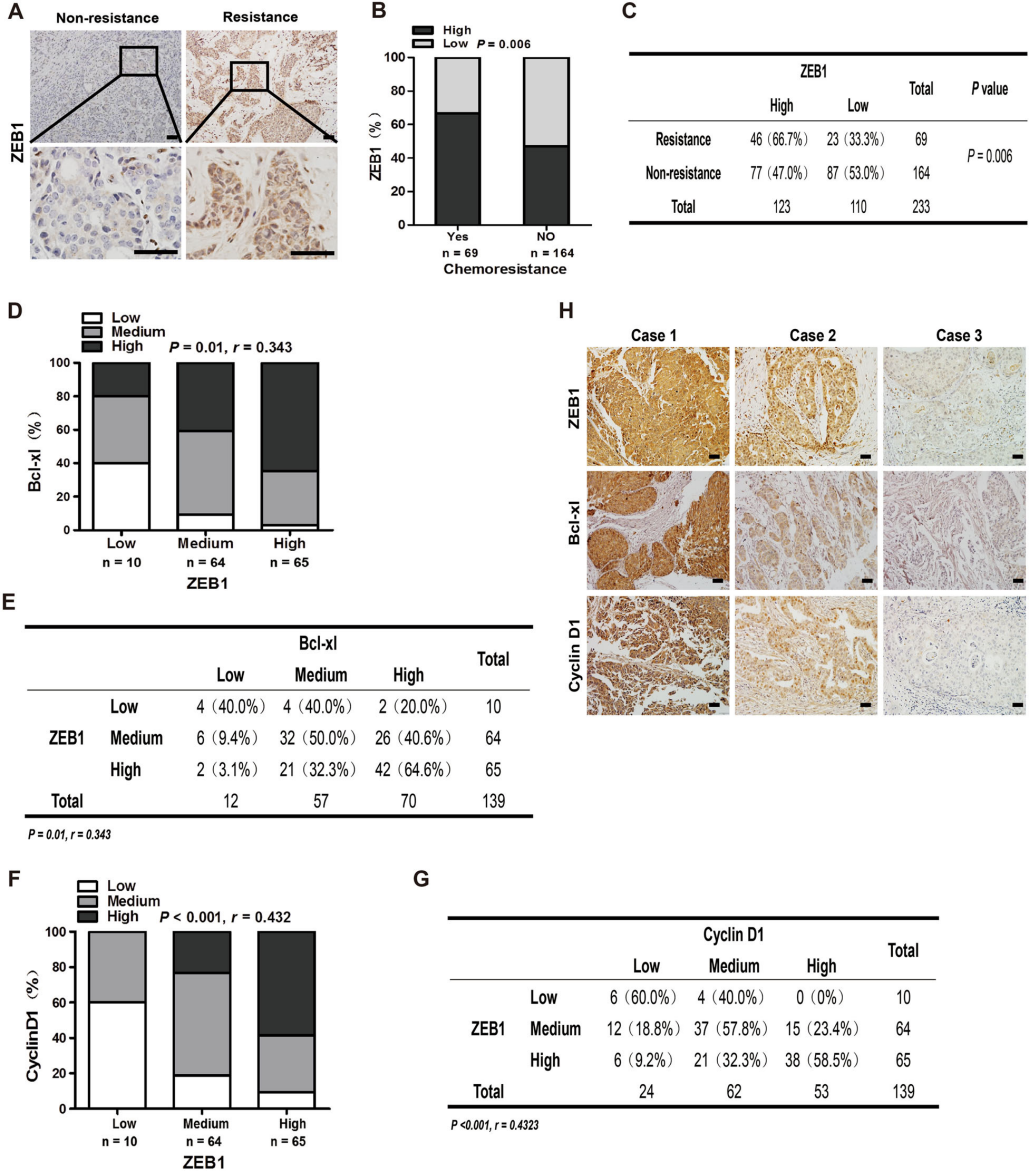
Elevated expression of ZEB1correlates with chemoresistance in human breast cancer **a** Representative images of immunohistochemical staining of ZEB1 in chemoresistant and non-resistant tumors are shown. Scale bars, 50 μm. **b**, **c** Expression of ZEB1 in chemoresistant tumors were significantly higher than that in non-resistant tumors. **d**, **e** The non-negative percentage analysis for Bcl-xL indicates a positive correlation with ZEB1 expression in primary breast cancer. **f**, **g** The non-negative percentage analysis for Cyclin D1 indicates a positive correlation with ZEB1 expression in primary breast cancer. **h** Representative images of immunohistochemical staining of ZEB1, Bcl-xL and cyclin D1 in tumors from three cases are shown. Scale bars, 50 μm

To assess the possible role of ZEB1 in chemoresistance, we performed immunohistochemical staining for ZEB1 in 233 cases of human breast cancer treated with anthracyclines-based neoadjuvant chemotherapy. The subjects were divided into two groups based on their responsiveness to the treatment and the criteria of response evaluation criteria in solid tumors. The results demonstrated that the expression of ZEB1 in chemoresistant tumors were significantly higher than that in non-resistant tumors (Fig. 1a–c). It has been reported that increased cell proliferation and disruption of apoptotic induction are key mechanisms that contribute to the failure of chemotherapy. B-cell lymphoma-extra large (Bcl-xL) and cyclin D1, which mediate anti-apoptotic response and cell proliferation, respectively, are upregulated in breast cancer tissues that are resistant to genotoxic drug treatment^37,38^. We further performed immunohistochemical staining for ZEB1, Bcl-xL and cyclin D1 in an independent cohort of 139 cases of primary breast carcinoma. The results demonstrated that the expression of ZEB1 was positively correlated with those of Bcl-xL and cyclin D1 (Fig. 1d–h), highlighting that increased expression of ZEB1 contributes to the cellular mechanisms that mediate breast cancer chemoresistance.

### ZEB1 regulates chemotherapeutic drug-induced DDR

**Fig. 2:**
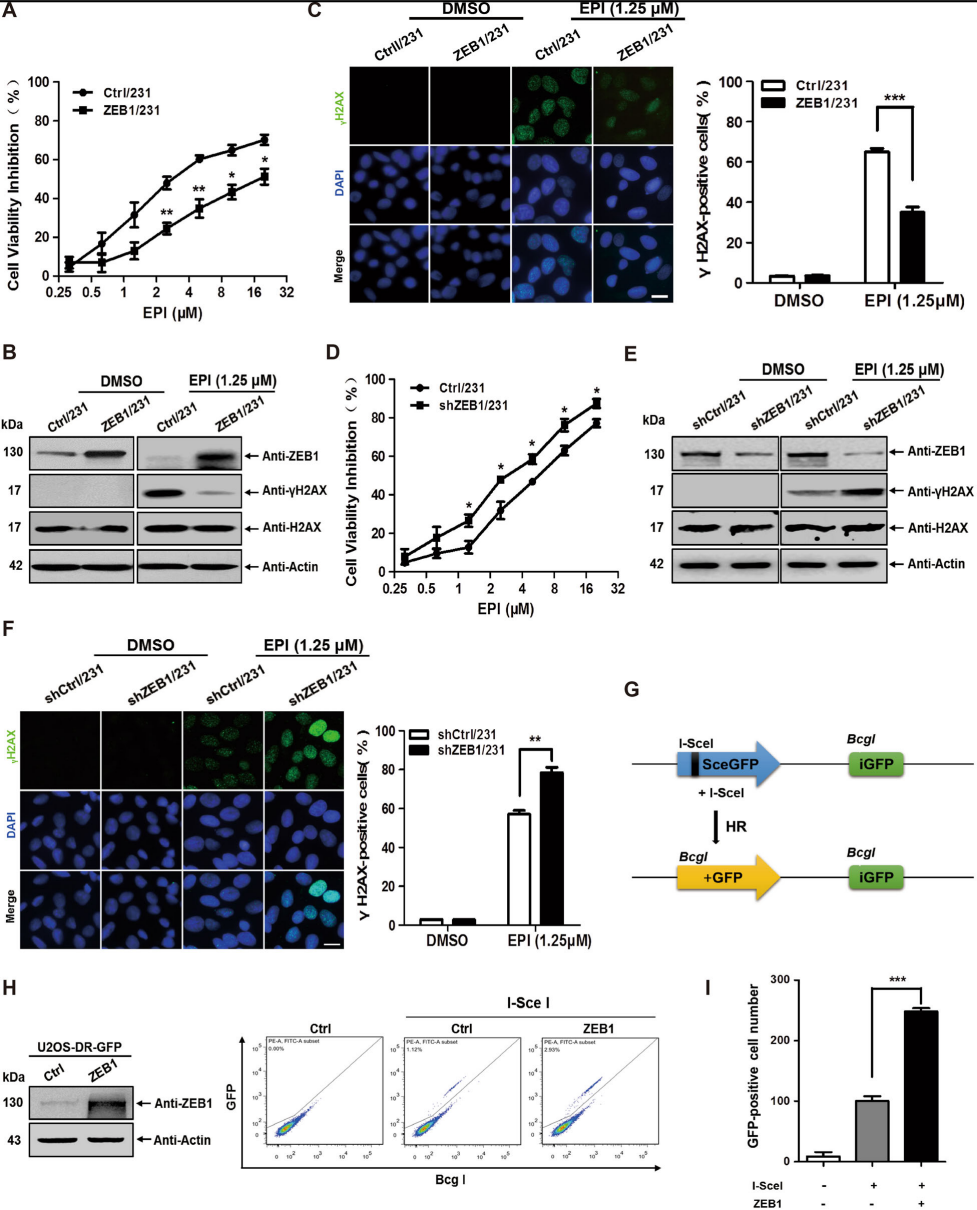
ZEB1 increases HR-mediated DNA damage repair in response to EPI treatment **a** ZEB1/231 and Ctrl/231 cells were treated with different concentrations of EPI for 48 h, respectively. EPI-induced cell growth inhibition was determined by cell viability assay. *P *<* 0.05, **P *<* 0.01 vs respective control in one-way ANOVA followed by Tukey’s honestly significant difference test. **b** EPI-induced expression of γH2AX protein was determined by immunoblotting and normalized to the levels of H2AX. Cropped blots are shown (full-sized blots are presented in Supplementary Fig. S9). **c** EPI-induced formation of γH2AX nucleic foci was measured by immunofluorescent staining. At least 500 nuclei were counted and the percentage of γH2AX-positive nuclei was determined. ***P *<* 0.001 vs respective control in Student’s *t* test. Scale bars, 20 μm. **d** shZEB1/231 and shCtrl/231 cells were treated with different concentrations of EPI for 48 h, respectively. EPI-induced cell growth inhibition was determined by cell viability assay. *P *<* 0.05 vs respective control in one-way ANOVA followed by Tukey’s honestly significant difference test. **e** EPI-induced expression of γH2AX protein was determined by immunoblotting and normalized to the levels of H2AX. Cropped blots are shown (full-sized blots are presented in Supplementary Fig. S10). **f** EPI-induced formation of γH2AX nucleic foci was measured by immunofluorescent staining. At least 500 nuclei were counted and the percentage of γH2AX-postitive nuclei was determined. **P *<* 0.01 vs respective control in Student’s *t* test. Scale bars, 20 μm. **g** The working model of HR repair in DR-GFP/U2OS cells is presented. **h** I-SceI endonuclease was introduced into ZEB1-expressiong and control DR-GFP/U2OS cells, respectively. HR repair assay was determined by flow cytometry. **i** The percentage of GFP-positive cells was quantified. ***P *<* 0.001 vs respective control in Student’s *t* test

To further determine the mechanism by which ZEB1 induces chemoresistance, ZEB1 was overexpressed or knocked down in MDA-MB-231 breast cancer cells using a lentiviral system (Supplementary Fig. S1). Cells were then treated with EPI or ETOP, and cell viability was measured. In response to EPI or ETOP treatment for 48 h, there was a significant decrease in cell growth inhibition in ZEB1/231 cells as compared to Ctrl/231 cells (Fig. 2a and Supplementary Fig. S2a). Furthermore, we examined the effect of ZEB1 on EPI- or ETOP- induced phosphorylation of histone H2A (γH2AX), which is a marker for DDR. The results confirmed that ZEB1 overexpression significantly reduced EPI- or ETOP- induced level of γH2AX (Fig. 2b and Supplementary Fig. S2b) and the formation of γH2AX nuclear foci (Fig. 2c and Supplementary Fig. S2c). Conversely, ZEB1 depletion resulted in the opposite effect in which it increased cell growth inhibition (Fig. 2d and Supplementary Fig. S2d) and enhanced γH2AX level and formation of γH2AX foci (Fig. 2e, f; Supplementary Fig. S2e and f). These results were not unique to MDA-MB-231 cells; ZEB1 overexpression in MCF-7 cells or ZEB1 knockdown in SUM-159 cells also reduced or enhanced EPI-induced γH2AX level and γH2AX nuclear foci, respectively (Supplementary Fig. S3). These results suggest that ZEB1 may play an important role for DNA damage repair and DSBs clearance in response to chemotherapy.

In mammalian cells, a key conserved pathway involved in DSBs repair is the HR-mediated repair pathway^8^. Thus, as seen Fig. 2g, we used a U2OS cell clone with chromosomal integration of a HR repair reporter consisting of two differentially mutated GFP genes (SceGFP and iGFP) oriented as direct repeats (DR-GFP). Expression of I-SceI endonuclease generates a site-specific DSBs in the SceGFP coding region, and when this DSBs is repaired by HR, the expression of GFP is restored and can be analyzed by flow cytometry to gauge the efficiency of HR repair^39,40^. We found that I-SceI expression led to an increase in the percentage of GFP-positive cells, indicating that I-SceI induced DSBs that are repaired by HR (Fig. 2h, i). Furthermore, upon I-SceI expression, ZEB1-expressing U2OS cells exhibited a significant increase (~2.5-fold) in the percentage of GFP-positive cells as compared to the control cells, demonstrating that ZEB1 enhanced DSBs repair by the HR-mediated pathway. These results collectively suggested that ZEB1 may play an important role for DNA damage repair and DSBs clearance in response to chemotherapy.

### Identification of ZEB1 target genes in breast cancer chemoresistance

**Fig. 3:**
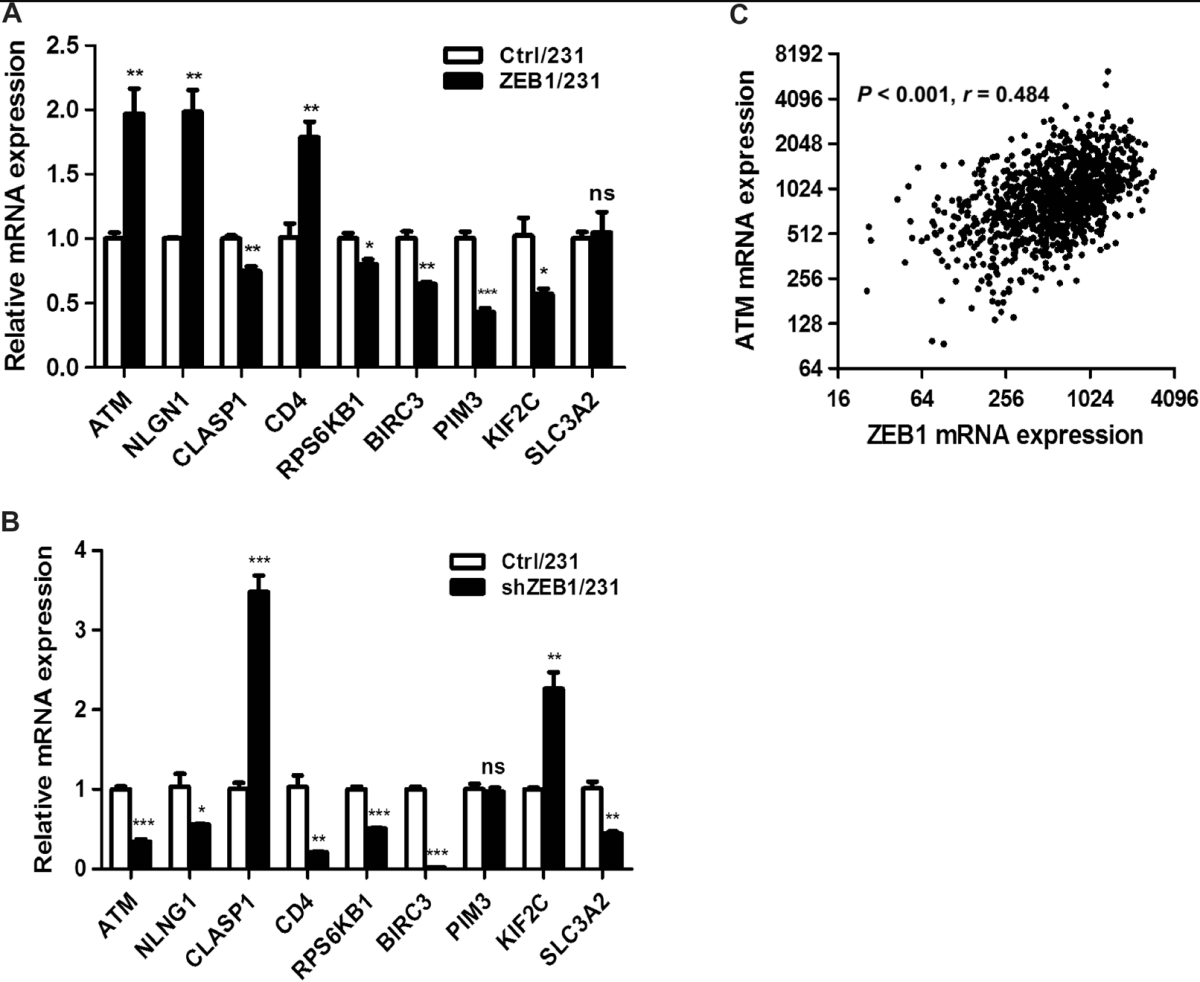
ZEB1 upregulates the expression ATM **a**, **b** The mRNA level of nine chemoresistance-related genes was examined by quantitative PCR (**a**) in ZEB1/231 vs Ctrl/231 cells and (**b**) in shZEB1/231 vs shCtrl/231 cells and normalized to the levels of β-actin. *P *<* 0.05, **P *<* 0.01, ***P *<* 0.001 vs respective control in Student’s *t* test. **c** TCGA database analysis indicates a positive correlation between ZEB1 and ATM mRNA levels. Statistical significance was determined by Spearman rank correlation analysis

Because ZEB1 functions as a DNA-binding protein with an essential role in breast cancer development^23,41^, we performed chromatin immunoprecipitation (ChIP) coupled deep DNA sequencing (ChIP-seq) to identify endogenous transcriptional targets of ZEB1 in MDA-MB-231 cells. We identified 147 genes bound by ZEB1 (Supplementary Fig. S4 and Table S1), nine of which have been reported to function in the regulation of drug resistance during tumorigenesis (Table 1). We then performed quantitative PCR to determine the correlation between ZEB1 and these chemoresistance-related genes in ZEB1/231 and shZEB1/231 cells. The results showed a significant positive correlation between ZEB1 and ATM expression at messenger RNA (mRNA) level, in that ectopic expression of ZEB1 increased, while ZEB1 interference reduced, ATM expression (Fig. 3a, b). Importantly, the correlation between ZEB1 and ATM, as well as the other seven target genes, were demonstrated by TCGA database analysis (Fig. 3c and Supplementary Fig. S5), highlighting a predominant role for ZEB1 in inducing ATM expression in breast cancer cells.

**Table 1:** Chemoresistance-related genes regulated by ZEB1

Gene	Fold-enrichment	Chromosome	Distance to TSS	ZEB1 consensus site	Validated by ChIP-PCR	Function	Reference(s)	mRNA correlation with ZEB1 in TCGA database
ATM	6.88	chr11:108108119–108108633	−849	CAGGTG	YES	Cell cycle checkpoint signaling pathways, DNA damage repair	Huizhen Sun et al., 2014	0.48
NLGN1	5.92	chr3:173601748–173602302	−184	CAGGTG	YES	Act as splicesite-specific ligands for beta-neurexins	Ben Davidson et al., 2014	0.38
CLASP1	8.67	chr2:122288282–122289026	2560	CAGGTG	YES	Regulation of microtubule dynamics	Katarzyna et al., 2014	0.29
CD4	8.11	chr12:6944779–6945395	−569	CAGGTG	YES	Initiate or augment the early phase of T-cell activation	Jee-eun kim et al., 2014	0.29
RPS6KB1	6.98	chr17:58045197–58045894	469	CAGGTG	YES	mTOR signaling pathway	Jun He et al., 2015	0.22
BIRC3	6.98	chr11:102191447–102191786	133	CAGGTG	YES	Inhibit apoptosis by binding toTRAF1 and TRAF2	Xu Chen et al., 2015	0.16
PIM3	8.67	chr22:50364008–50364825	−401	CAGGTG	YES	Cell proliferation and survival, coexpression with MYC	Dapeng Xu et al., 2013	−0.30
KIF2C	4.74	chr1:45186733–45187724	−507	CAGGTG	NO	Microtubule-dependent molecular motors	Fung Zhao et al., 2014	−0.37
SLC3A2	10.31	chr11:62608316–62609696	1347	CAGGTG	NO	Regulation of intracellular calcium levels, transports L-type amino acids	Zunyan Dai et al., 2007	−0.40

### ZEB1 induces ATM expression by stimulating its transcriptional activity

**Fig. 4:**
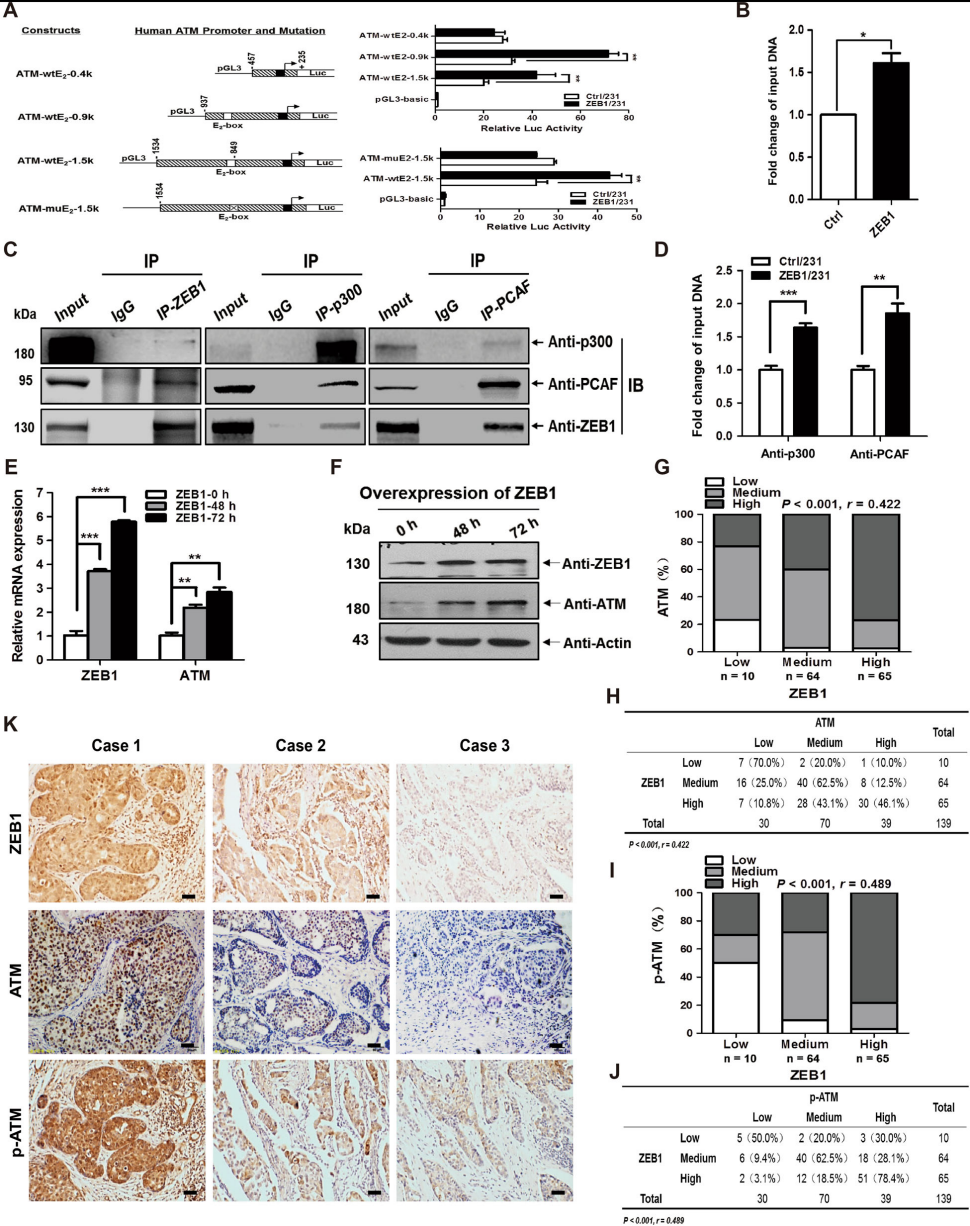
ZEB1 induces ATM expression by stimulating transcription of the ATM gene **a** MDA-MB-231 cells were co-transfected with the ZEB1 expression plasmid and different wild-type ATM promoter luciferase reporter constructs. Extract luciferase activities were determined 36 h after transfection using a Betascope analyzer. Luciferase values were normalized to Renilla activities. **P < 0.01 vs respective control in Student’s *t* test. **b** Overexpression of ZEB1 significantly enhanced its recruitment to the endogenous ATM promoter as confirmed by a quantitative ChIP assay. *P < 0.05 vs respective control in Student’s *t* test. **c** The interactions among ZEB1, p300 and PCAF protein were analyzed by co-immunoprecipitation in ZEB1/231 cells. Cropped blots are shown (full-sized blots are presented in Supplementary Fig. S11). **d** Overexpression of ZEB1 significantly enhanced the recruitment of p300 and PCAF to the endogenous ATM promoter as confirmed by a quantitative ChIP assay. **P < 0.01, ***P < 0.001 vs respective control in Student’s *t* test. **e**, **f** MDA-MB-231 cells were transiently transfected with the ZEB1 expression plasmid or vector control. At the indicated time points, expression of ZEB1 and ATM were verified by (**e**) quantitative PCR and (**f**) immunoblotting and normalized to the levels of β-actin. Cropped blots are shown (full-sized blots are presented in Supplementary Fig. S12). **P *<* 0.01, ***P *<* 0.001 vs respective control in one-way ANOVA followed by Tukey’s honestly significant difference test. **g**, **h** The non-negative percentage analysis for ATM indicates a positive correlation with ZEB1 expression in primary breast cancer. **i**, **j** The non-negative percentage analysis for p-ATM indicates a positive correlation with ZEB1 expression in primary breast cancer. **k** Representative images of immunohistochemical staining of ZEB1, ATM, and p-ATM in tumors from three cases are shown. Scale bars, 50 μm

Our ChIP-seq analysis revealed that ZEB1 directly binds to the ATM promoter, suggesting that ZEB1 may induce the transcription of ATM. Consequently, we performed promoter-reporter assays to examine the regulation of ATM transcription by ZEB1. As shown in Fig. 4a, the wild-type −1534/+235 promoter of the ATM gene has one canonical E_2_-box element (CAGGTG) at position −854/−849, to which ZEB1 can potentially bind in our ChIP-seq analysis^41^. The result of luciferase assay indicated that ZEB1 overexpression increased the promoter activity of ATM-wtE_2_-1.5k reporter by ~2.1-fold relative to the control without ZEB1 transfection in MDA-MB-231 cells (Fig. 4a). A series of truncated and mutated ATM promoter-reporter constructs were then generated for analysis. The results showed that deletion or site-directed mutagenesis of the E_2_-box element was sufficient to abolish ZEB1-activated transcription of the ATM promoter. Importantly, quantitative ChIP assay indicated that ZEB1 overexpression resulted in a 1.7-fold increase in its binding to the E_2_-box element in the endogenous ATM promoter (Supplementary Fig. S6a and Fig. 4b). Mechanistically, p300 and PCAF have been shown to act as cofactors for ZEB1, which can reverse its suppressive effects on gene transcription^42^. We thus investigated the interaction between ZEB1 and p300/PCAF by co-immunoprecipitation. The results demonstrated that both p300 and PCAF were co-immunoprecipitated with ZEB1 in ZEB1/231 cells (Fig. 4c). The ChIP experiments further revealed that both p300 and PCAF were recruited to the ATM promoter in an E_2_-box-dependent manner, which was further increased by ZEB1 overexpression (Supplementary Fig. S6b and Fig. 4d). The observations collectively suggested that ZEB1 activates ATM transcription by forming a ZEB1/p300/PCAF complex on the ATM promoter.

We then substantiated ZEB1-regulated expression of ATM at mRNA and protein levels and in human breast cancer. As shown in Fig. 4e, a significant, time-dependent upregulation of ATM mRNA was observed in ZEB1-expressing MDA-MB-231 cells. Western blotting assay further confirmed ZEB1-induced expression of ATM at protein level (Fig. 4f). In contrast, ZEB1 depletion resulted in the opposite effect, which downregulated ATM mRNA and protein expression (Supplementary Fig. S7a and b). Moreover, ZEB1-induced ATM expression was demonstrated in ZEB1-expressing MCF-7 and ZEB1-interfering SUM-159 cells (Supplementary Fig. S7c and d). To determine the correlation between ZEB1 and ATM expression in human breast cancer, we performed immunohistochemical staining in 139 cases of primary breast carcinoma. We found that samples displaying a high percentage of ZEB1-positive cells exhibited a high level of ATM expression (Fig. 4g, h), whereas cancers exhibiting lower ZEB1 levels showed diminished ATM expression. Importantly, the results also confirmed a positive correlation between the expression of ZEB1 and phosphorylated ATM (p-ATM) (Fig. 4i, j). Immunohistochemical staining of samples from three representative subjects confirmed the positive relationship between ZEB1, ATM and p-ATM expression (Fig. 4k), which is consistent with our finding that ZEB1 activates ATM in breast cancer cells and may thus promote DNA damage repair in response to chemotherapy.

### ZEB1 renders breast cancer chemoresistance by targeting ATM

**Fig. 5:**
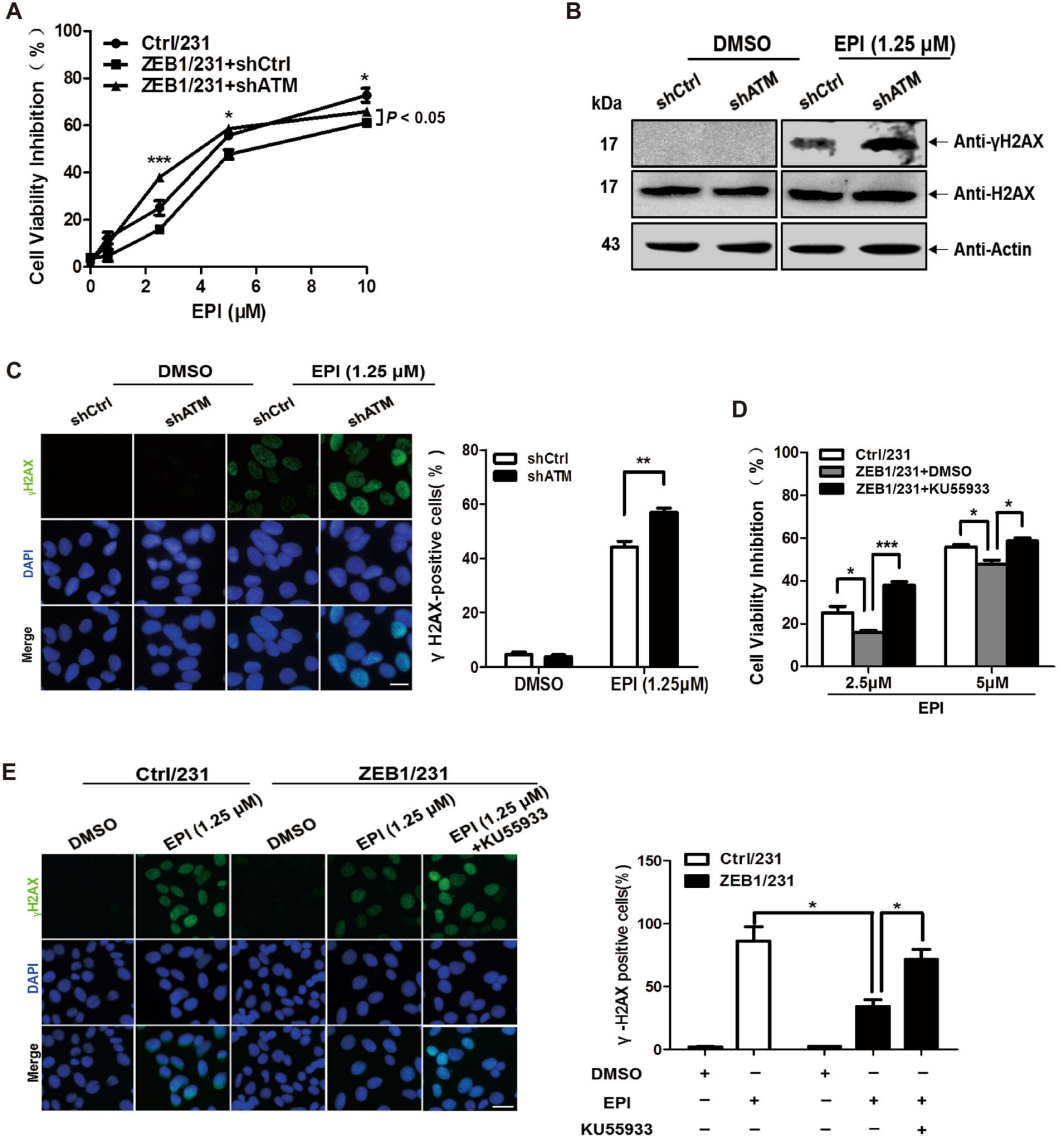
ATM is required for ZEB1-mediated chemoresistance **a**–**c** The specific shRNA targeting ATM or scramble shRNA was introduced into ZEB1/231 cells, followed by treatment with different concentrations of EPI for 48 h. **a** Cell growth inhibition was determined by cell viability assay. *P *<* 0.05, ***P *<* 0.001 vs respective control in one-way ANOVA followed by Tukey’s honestly significant difference test. **b** EPI-induced expression of γH2AX protein was determined by immunoblotting and normalized to the levels of H2AX. Cropped blots are shown (full-sized blots are presented in Supplementary Fig. S13). **c** EPI-induced formation of γH2AX nucleic foci was measured by immunofluorescent staining. At least 500 nuclei were counted and the percentage of γH2AX-postitive nuclei was determined. **P *<* 0.01 vs respective control in Student’s *t* test. Scale bars, 20 μm. **d**, **e** ZEB1/231 cells were treated with 10 μM KU-55933, followed by treatment with the indicated concentrations of EPI for 48 h. EPI-induced cell viability inhibition (**d**) and formation of γH2AX nucleic foci (**e**) were determined. *P *<* 0.05, ***P *<* 0.001 vs respective control in Student’s *t* test

Next, we tested whether ZEB1/ATM axis would functionally confer resistance to genotoxic drug-mediated cell growth inhibition in MDA-MB-231 cells. Thus, a control- or ATM-targeted short hairpin RNA (shRNA) was introduced into ZEB1/231 cells, followed by treatment with EPI. Knockdown of ATM was assessed by western blotting (Supplementary Fig. S8a). Cell viability assays indicated that ectopic expression of ZEB1 led to a decreased growth inhibition in response to EPI, which was attenuated by ATM knockdown (Fig. 5a). EPI-induced level of γH2AX and formation of γH2AX foci were further measured in ATM-depleted ZEB1/231 cells. The results demonstrated that ATM depletion abolished the ability of ZEB1 to suppress EPI-induced γH2AX level (Fig. 5b) and formation of γH2AX nuclear foci (Fig. 5c). Similarly, ZEB1/231 cells were pre-incubated with an ATM kinase inhibitor, KU-55933, followed by treatment with EPI. The results confirmed that inhibition of ATM activity attenuated ZEB1-decreased cell viability inhibition (Fig. 5d) and formation of γH2AX foci (Fig. 5e) in response to EPI. Same results were obtained in ETOP-treated ZEB1/231 cells as in the EPI-treated cells (Supplementary Fig. S8b and c). Taken together, we have found an important mechanism that ZEB1 regulates the sensitivity of breast cancer cells to genotoxic drug treatment in an ATM-dependent manner.

### ZEB1 promotes breast cancer chemoresistance in vivo

**Fig. 6:**
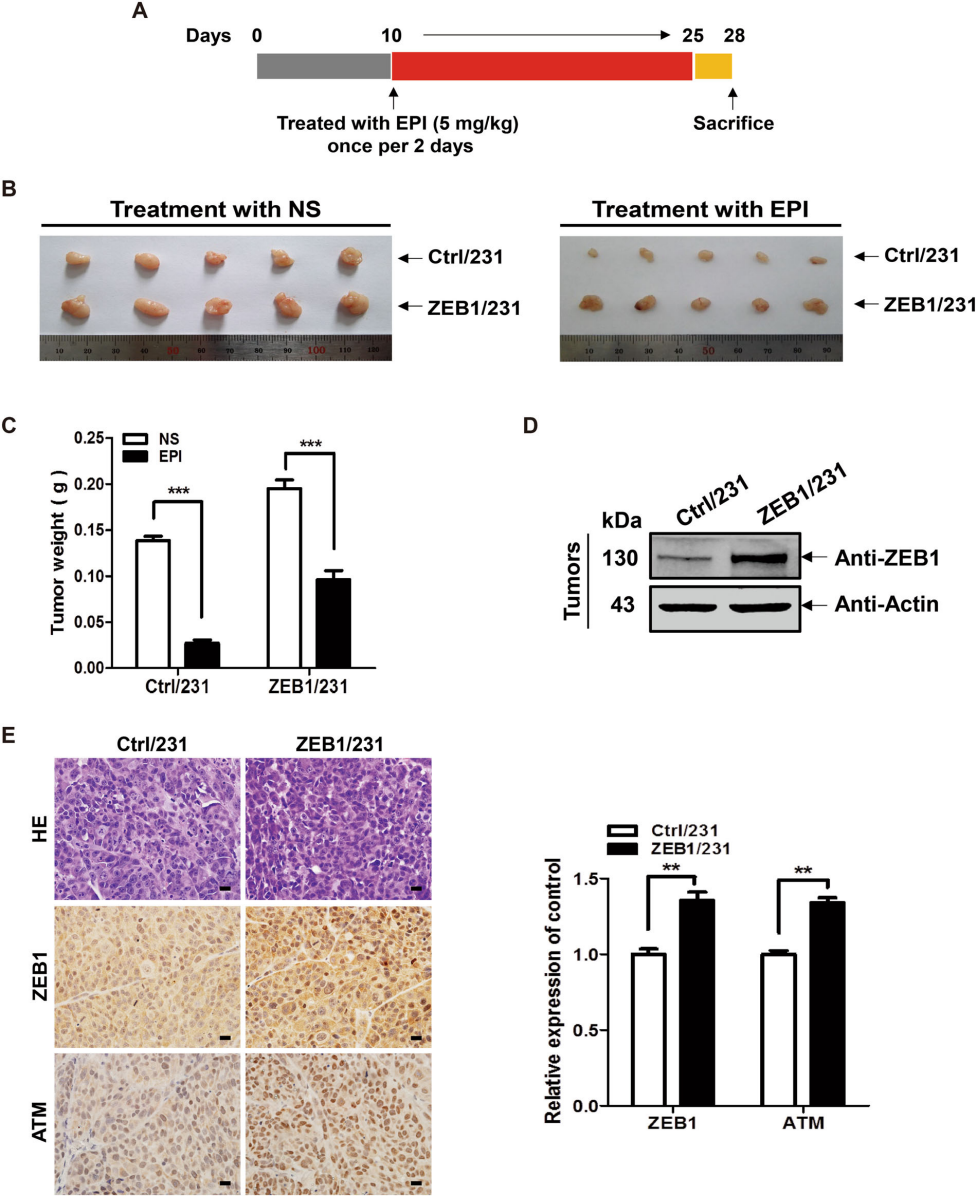
Elevated expression of ZEB1 promotes chemoresistance in vivo in a nude mouse xenograft model **a** A total of 1.5 × 10^6^ ZEB1/231 and Ctrl/231 cells were respectively injected into the mammary fat pads of nude mice (*n* = 5). Tumor development was allowed for 10 days, and then the mice were intraperitoneally injected with 5 mg/kg EPI (once per 2 days) for another 2 weeks. **b** Tumors from ZEB1/231 and Ctrl/231 mice that were, respectively, treated with EPI and normal saline (NS) are shown. **c** Approximate tumor weights were measured. ***P *<* 0.001 vs respective control in Student’s *t* test. **d**, **e** Expression of ZEB1 and ATM in breast cancer xenografts was examined by (**d**) western blotting and (**e**) immunohistochemical staining. Cropped blots are shown (full-sized blots are presented in Supplementary Fig. S14). **P *<* 0.01 vs respective control in Student’s *t* test. Scale bars, 20 μm

Next, we determined whether elevated ZEB1 expression in breast cancer cells influences tumor response to chemotherapeutic treatment in vivo. ZEB1/231 or Ctrl/231 cells were injected into the mammary fat pads of female BALB/c nude mice to establish a nude mouse xenograft model, followed by treatment with EPI (Fig. 6a). The results indicated that EPI effectively inhibited tumor growth in BALB/c nude mice with control tumors but not in the mice with ZEB1-expressing tumors (Fig. 6b). Upon treatment with EPI, tumor weights were significantly higher in mice injected with ZEB1/231 cells compared with those injected with Ctrl/231 cells (Fig. 6c). Western blotting and immunohistochemical staining further confirmed the upregulation of ZEB1 and ATM expression in tumors from ZEB1/231 mice as compared to the Ctrl/231 mice (Fig. 6d, e). Collectively, these data demonstrate that ZEB1 can reduce breast cancer chemosensitivity in vivo.

## Discussion

Chemotherapy plays an important role in breast cancer management, and one of the main barriers in curing breast cancer is intrinsic and therapy-induced chemoresistance. To overcome this obstacle, it is important to identify the critical determinants of chemoresistance and to develop safe and effective tumor chemosensitizers^3,4^. Our work reveals a key role for ZEB1 in breast cancer chemoresistance. First, we showed that patients with tumors that express high levels of ZEB1 have a weaker response to chemotherapy. Second, we found that ZEB1 induces ATM activity by forming a ZEB1/p300/PCAF complex on the ATM promoter. Expression of ZEB1 is increased in breast cancer and positively correlates with ATM protein levels. Third, ZEB1 promotes DDR in breast cancer cells in response to chemotherapy and confers chemoresistance by reducing genotoxic drug-induced DSBs in an ATM-dependent manner. Finally, downregulation of ZEB1 increases the sensitivity of breast cancer cells to chemotherapy in vitro and in vivo. Therefore, our study reveals the possibility that ZEB1 acts as a determinant of chemoresistance in breast cancer.

Our data suggest that patients are less likely to benefit from genotoxic drug-based neoadjuvant chemotherapy if they have breast cancers with high ZEB1 expression, compared with tumors with low ZEB1 expression. It is known that ATM kinase is constitutively activated in chemoresistant tumor cells^43–46^, which is consistent with our results showing that ZEB1 induces ATM activation by recruiting the transcriptional coactivators p300 and PCAF to the ATM promoter. By examining 139 primary breast cancer specimens, we found that ZEB1 expression is elevated in cancer tissues, and this expression positively correlates with the expression of ATM and p-ATM, suggesting that increased ZEB1 expression may be the primary cause of hyperactivated ATM in some breast cancers. Taken together with our observations that ZEB1 overexpression decreases breast cancer cell sensitivity to EPI and ETOP treatment in an ATM-dependent fashion, ATM upregulation may at least partially account for the resistance of this subset of breast cancers against chemotherapy. These findings have shed new light on the mechanisms of chemoresistance in breast cancer.

A growing body of evidence suggests that EMT regulators play important roles in the acquisition of chemoresistance and radioresistance during tumor development^47–50^. For example, Zhang et al.^49^ reported that ATM-mediated stabilization of ZEB1 promotes DDR and radioresistance through CHK1. Here, we extended the study to identify ZEB1 as an ATM regulator, indicating a positive feedback loop between ZEB1 and ATM regulation: in response to genotoxic treatment, ATM kinase is activated, which phosphorylates and stabilizes ZEB1; ZEB1 in turn interacts with p300 and PCAF, a process that upregulates ATM expression. In fact, it has been shown that ZEB1 recruits distinct transcriptional cofactors in different contexts^42,51–53^. For example, Postigo et al.^42^ reported that ZEB1 normally recruits the corepressor CtBP1 and not coactivators p300 and PCAF; however, in response to TGF-β, recruitment of p300/PCAF displaces CtBP1 from ZEB1, which then activates TGF-β-responsive genes.

It is known that ZEB1 promotes cancer progression through a variety of genetic and epigenetic mechanisms^27–33,54–56^. Our research reveals that one of these mechanisms is to promote DDR by inducing ATM expression, thus conferring resistance to chemotherapeutic drugs. Consistent with our results that ZEB1 overexpression attenuates EPI- and ETOP-induced cell growth inhibition, ZEB1 has been shown to facilitate cancer cell proliferation in the presence of DNA damage^57,58^. Further supporting our conclusion, ZEB1 enhances HR-mediated repair of DSBs in response to radiation^49^. As HR is an error-free repair mechanism for DSBs, ZEB1 may therefore function to maintain genome integrity. This new function of ZEB1 may appear to be inconsistent with the current knowledge that cancer cells are commonly associated with genome instability and that many oncogenes promote, rather than maintain, genome instability^59,60^. However, we reason that a certain level of genome integrity is needed in order for cancer cells to continue proliferating. ZEB1 may thus function as an oncogene that maintains a balance to allow cancer cells to proliferate in the presence of genome instability that serves as a source of cancer-promoting mutations, without accumulation of excessive DNA damage that leads to proliferative arrest or apoptosis. This function of ZEB1 in promoting DDR is especially important for the survival and proliferation of cancer cells in the face of chemotherapeutic agents that induce massive DNA damage, which leads to chemoresistance. Intriguingly, the fact that ZEB1-mediated attenuation of cell cycle arrest in EPI- and ETOP-treated breast cancer cells is not associated with an enhancement of cytotoxicity supports a role of ZEB1 in facilitating the repair of DNA lesions^61,62^.

ZEB1 overexpression has been observed in human breast cancer^54^ and several other cancers^27–33^. Because depletion of ZEB1 chemosensitizes breast cancer cells in vitro and in vivo, we suggest that ZEB1-targeting agents have the potential to be used as tumor chemosensitizers. Moreover, various ATM inhibitors are being tested in anti-cancer treatment^63^, which warrant investigation as candidate chemosensitizing agents for breast cancer with high levels of ZEB1.

## Materials and methods

### Tissue samples

A total of 233 breast cancer subjects received anthracyclines-based neoadjuvant chemotherapy were obtained from the General Hospital of the People’s Liberation Army (Beijing, China) along with pathologic information (Table S2). Besides, 139 samples of primary breast cancer in tissue array were obtained from Alenabio Biotechnology Ltd., Xi’an, China. All patients had histologically confirmed invasive ductal carcinoma of breast cancer. This study was approved by the institutional ethics committees at PLAGH and Medical College of Nankai University. All patients provided informed consent according to the latest version of the Helsinki Declaration on human research ethics. All methods were carried out in accordance with the approved guidelines.

### Immunohistochemical analysis

Immunohistochemical analysis of paraffin-embedded sections was performed using the Envision Kit (Dako) following the manufacturer’s protocols. Sections were boiled in retrieval solutions to expose antigens. The specific antibodies (Supplementary Information) were applied to the sections. Normal rabbit IgG was used as a control (CST, #2729). Slides were counterstained with hematoxylin, dehydrated, and mounted. Immunostaining was independently evaluated by two pathologists.

### Cell culture and transfection

Human breast cancer cell lines MDA-MB-231, MCF-7 and SUM-159 were maintained in Dulbecco’s modified Eagle’s medium (DMEM) supplemented with 10% FBS, 100 IU penicillin and 100 mg/ml streptomycin. Human osteosarcoma cell line U2OS-DR-GFP were maintained in RPMI 1640 supplemented with 10% FBS, 100 IU penicillin and 100 mg/ml streptomycin. Cells were transfected using Lipofectamine 2000 (Invitrogen) following the manufacture’s protocol.

### Plasmid construction

The human complementary DNAs (cDNAs) fragment encoding the full-length ZEB1 sequence^41^ was prepared by PCR and cloned into pLV-EF1-MCS-IRES-Bsd (Biosettia). The lentiviral-based vector pLV-H1-EF1α-puro (Biosettia) was used to express shRNAs in breast cancer cells. The human ATM promoter (−1534/+235) sequences were obtained by PCR from human genomic DNA and cloned into pGL3‑basic vector (Promega). Mutagenesis of the E_2_‑box in the human ATM promoter was performed using a QuikChange Site‑Directed Mutagenesis kit (Stratagene).

### Generation of lentivirus system

Lentiviruses were generated by transfecting subconfluent HEK293T cells together with the lentiviral vectors and packaging the plasmids by calcium phosphate transfection. Viral supernatants were collected 48 h after the transfection, centrifuged at 75,000×*g* for 90 min, resuspended and filtered through 0.45-μm filters (Millipore).

### Cell proliferation assay

Cells were seeded onto a 96-well plate at a density of 4 × 10^3^ cells per well, followed by treatment with different concentrations of EPI for 48 h. Cell viability was then assessed using the CCK-8 assay according to the manufacturer’s protocols (Dojindo). Six parallel replicates were measured for each sample.

### RNA extraction and quantitative RT-PCR

Cells were transfected with ZEB1 expression plasmid or ZEB1-targeted shRNA. Total RNA (0.5 μg) from each sample was collected using TRIzol reagent (Invitrogen) and was used for first-strand cDNA synthesis using M-MLV Reverse Transcriptase (Takara). The specific products of ZEB1 and ATM were amplified by quantitative PCR using TransStart Green Q-PCR SuperMix Kit (TransGen). GAPDH was used as a normalization control.

### Immunoblotting assay

Preparation of total cell extracts and immunoblotting with appropriate antibodies was performed as previously described^41^. The appropriate antibodies were used as seen in Supplementary Information. Labeled proteins were visualized by an ECL chemiluminescence kit (Millipore).

### Immunofluorescence microscopy

Cells were washed twice with PBS and fixed in 4% paraformaldehyde. The cells were incubated with rabbit polyclonal Ab against γH2AX (ab2839, Abcam) for 3 h at 37 °C, washed with PBS and incubated with DyLight 488-conjugated secondary antibody (Cwbiotech) for another 3 h. The cells were stained with DAPI (50 μg/ml) for 5 min for the detection of nuclei by Confocal FV1000 (Olympus). γH2AX-positive cell was calculated with (γH2AX add-in cells/DAPI stained cells) × 100%. At least 500 cells were counted per well.

### HR DNA repair assay

A U2OS derivative clone stably expressing HR reporter DR-GFP was described previously^39,40^. U2OS-DR-GFP cells were seeded onto a six-well plate at a density of 5 × 10^5^ cells per well, followed by co-transfection with pCBASce (the I-SceI expression plasmid) and ZEB1 expression plasmid. At 72 h after transfection, the number of GFP-positive cells was measured by flow cytometric analysis using a LSR Fortessa (BD).

### The Cancer Genome Atlas database analysis

Analyses of The Cancer Genome Atlas (TCGA) database were performed on primary breast cancer tumor samples with RNA-sequencing data. Level 3-normalized gene expression (RNA Seq V2) was obtained from cBioPortal (http://www.cbioportal.org/). A total of 1098 breast invasive carcinoma patients were analyzed. The gene expression data is transformed into log2 scale.

### Luciferase assay

Cells were co-transfected with the wild-type or mutant human ATM promoters and ZEB1 expression plasmid in 24-well plates. Lysates were prepared at 36 h after transfection, and luciferase activities were measured using the dual-luciferase reporter assay system (Promega) according to the manufacturer’s protocols. The luciferase activities were normalized to the values for Renilla luciferase.

### Immunoprecipitation assay

Cell lysates was incubated with specific antibodies plus Protein G agarose beads (Invitrogen) at 4 °C overnight, followed by three washes with a buffer containing 50 mM Tris (pH 7.5), 100 mM NaCl, 7.5 mM EGTA, and 0.1% Triton X-100. The antibodies used for immunoprecipitation were shown in Supplementary Information.

### Chromatin immunoprecipitation

ChIP assays were performed using an EZ-ChIP kit (Millipore) according to the manufacturer’s instructions. The antibodies used in these experiments were shown in Supplementary Information. The fragment of human ATM promoter containing the E_2_-box element in immunoprecipitates was amplified by quantitative PCR.

### Tumor xenograft experiments

Cells were collected and suspended in 200 μl of PBS at a concentration of 5 × 10^6^ cells per ml, then injected into the mammary fat pads of female BALB/c nude mice. Tumor development was allowed for 10 days. The mice were then intraperitoneally injected with 5 mg/kg EPI (once per 2 days) for another 2 weeks. The mice were killed when tumor masses were detected 4 weeks after surgery. Tumor tissues were also processed and sectioned for histological evaluation.

The study protocol was approved by the Ethics Committee of Medical College of Nankai University. The animal experiments were performed in strict accordance with the National Institutes of Health Guidelines for the Care and Use of Laboratory Animals. Mice were sacrificed under anesthesia (10% chloral hydrate, peritoneal injection), and all efforts were made to minimize discomfort and pain.

### Statistical analysis

Statistical analyses were performed using SPSS 13.0 software, the data from all the experiments are presented as means ± SD and represent three independent experiments. One-way analysis of variance was used to compare means between treatment groups. Where appropriate, Student’s *t* test for unpaired observations was applied. A *P* value <0.05 was considered significant. The *r*-value test was used to evaluate correlation analysis.

## Electronic supplementary material

Supplementary Material
