# Supplementary material for: ZEB1 confers chemotherapeutic resistance to breast cancer by activating ATM
Source: Cell Death Dis. 2018 Jan 19;9(2):57. doi: 10.1038/s41419-017-0087-3 (PMC5833408; doi:10.1038/s41419-017-0087-3)
Supplement: Supplementary file 1 — Supplementary Material [file 41419_2017_87_MOESM1_ESM.docx]

**ZEB1 confers chemotherapeutic resistance to breast cancer by activating ATM**

Xiang Zhang^1^, Zhen Zhang^2^, Qing Zhang^2^, Quansheng Zhang^3^, Peiqing Sun^4^, Rong Xiang^2^, Guosheng Ren^1^, Shuang Yang^2,^*

^1^Chongqing Key Laboratory of Molecular Oncology and Epigenetics, The First Affiliated Hospital of Chongqing Medical University, Chongqing 400016, China

^2^Tianjin Key Laboratory of Tumor Microenvironment and Neurovascular Regulation, Medical College of Nankai University, Tianjin 300071, China

^3^Tianjin Key Laboratory of Organ Transplantation, Tianjin First Center Hospital, Tianjin 300192, China

^4^Department of Cancer Biology, Wake Forest University School of Medicine, Winston-Salem, NC 27157, USA

*Corresponding author: Shuang Yang, Medical College of Nankai University, 94 Weijin Road, Tianjin 300071, China, Tel: +86-22-23509557, Fax: +86-22-23505501, E-mail: [yangshuang@nankai.edu.cn](mailto:yangshuang@nankai.edu.cn)

**Supplementary materials and methods**

*Primers*

| Construction of full-length ZEB1 expression vector | |
| --- | --- |
| forward | 5’-ATGGCGGATGGCCGCC‑3’ |
| reverse | 5’-TTAGGCTTCATTTGTCTTTTCTTCA‑3’ |
| Construction of shRNA vector | |
| shZEB1-1 | 5’-CGGCGCAATAACGTTACAAAT‑3’ |
| shZEB1-2 | 5’-GGCGCAATAACGTTACAAA‑3’ |
| shZEB1-3 | 5’-CCTCTCTGAAAGAACACATTA‑3 |
| shATM-1 | 5’-TCAAGAACACCACTTCGCT‑3’ |
| shATM-2 | 5’-TCAAGAACACTCAGCTCCCT‑3’ |
| Construction of ATM promoter | |
| wt 1.5k forward | 5’-ACCGAAACTCAACTAGATTACC‑3’ |
| wt 0.9k forward | 5’-CATTTCACACCTCTACACTGG‑3’ |
| wt 0.4k forward | 5’-CCTCAAAGGTCCTTCTGTCC‑3’ |
| wt reverse | 5’-CAAACCCTGCGTGACTGC‑3’ |
| Site-Directed Mutagenesis of ATM promoter | |
| forward | 5’-CTTTCCTTCCGAATCCGCCC**CATATG**TTCCACCCCGAGCTTCCCC‑3’ |
| reverse | 5’-GGGGAAGCTCGGGGTGGAA**CATATG**GGGCGGATTCGGAAGGAAAG‑3’ |
| Quantitative RT-PCR | |
| human ZEB1 forward | 5’-ATGTGGCTAGTTTGTCCTC-3’ |
| human ZEB1 reverse | 5’-AGCAAGATTTCCTCCAGGTC-3’ |
| human ATM forward | 5’-GGCTATTCAGTGTGCGAGACA-3’ |
| human ATM reverse | 5’-TGGCTCCTTTCGGATGATGGA-3’ |
| Quantitative ChIP | |
| forward | 5’-CAGACAAATGCTCTTTCAGGGG-3’ |
| reverse | 5’-GCCTCTTTGTATGTAGATGCTC-3’ |

*Antibodies*

| Marker | Species | Application | Manufacturer | Catalog No. | Dilution |
| --- | --- | --- | --- | --- | --- |
| anti-ZEB1 | Rabbit | IHC | Abcam | ab87280 | 1:100 |
|  | Rabbit | IB | Santa Cruz | sc-25388 | 1:1000 |
|  | Rabbit | CHIP | Proteintech | 221544-1-AP | 1:1000 |
| anti-ATM | Rabbit | IHC | Abcam | ab183324 | 1:100 |
|  | Rabbit | IB | Abcam | ab32420 | 1:1000 |
| anti-ATM (phopho S1981) | Rabbit | IHC | Abcam | ab81292 | 1:500 |
| anti-Bcl-xL | Rabbit | IHC | CST | 2764 | 1:100 |
| anti-Cyclin D1 | Rabbit | IHC | CST | 2978 | 1:100 |
| anti-H2AX | Rabbit | IB | Abcam | ab131492 | 1:1000 |
| anti-γH2AX | Rabbit | IB | Abcam | ab2839 | 1:1000 |
|  |  | IF |  |  | 1:200 |
| anti-p300 | Mouse | IP | Abcam | ab3164 | 2 μg/reaction |
|  |  | CHIP |  |  | 1 μg/reaction |
|  |  | IB |  |  | 1:200 |
| anti-PCAF | Rabbit | IP | Abcam | ab2188 | 2 μg/reaction |
|  |  | CHIP |  |  | 1 μg/reaction |
|  |  | IB |  |  | 1:500 |
| anti-Snail | Rabbit | IB | CST | C15D3 | 1:1000 |
| anti-Twist | Mouse | IB | Abcam | ab175430 | 1:1000 |
| anti-Vimentin | Rabbit | IB | CST | D21H3 | 1:1000 |
| anti-N-Cadherin | Mouse | IB | BD | 610920 | 1:1000 |
| anti-Ki-67 | Rabbit | IHC | Abcam | ab16667 | 1:200 |
| anti-actin | Mouse | IB | Santa Cruz | sc-47778 | 1:1000 |

IHC: Immunohistochemistry; IB: Immunoblotting; IF: Immunofluorescence; IP: Immunoprecipitation; ChIP: Chromatin immunoprecipitation


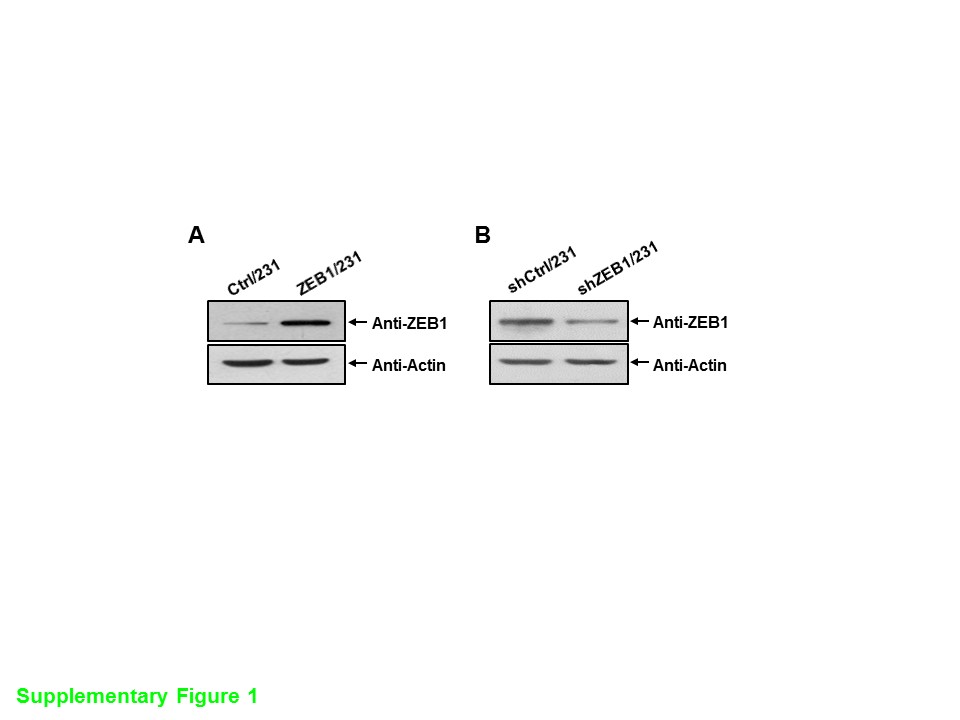


Figure S1 Gain- or loss-of-function of ZEB1 in MDA-MB-231 cells. (**a,b**) MDA-MB-231 cells were stably transfected with the ZEB1 expression plasmid (**a**) or specific shRNA targeting ZEB1 (**b**). The expression of ZEB1 was verified by immunoblotting and normalized to the levels of β-actin.


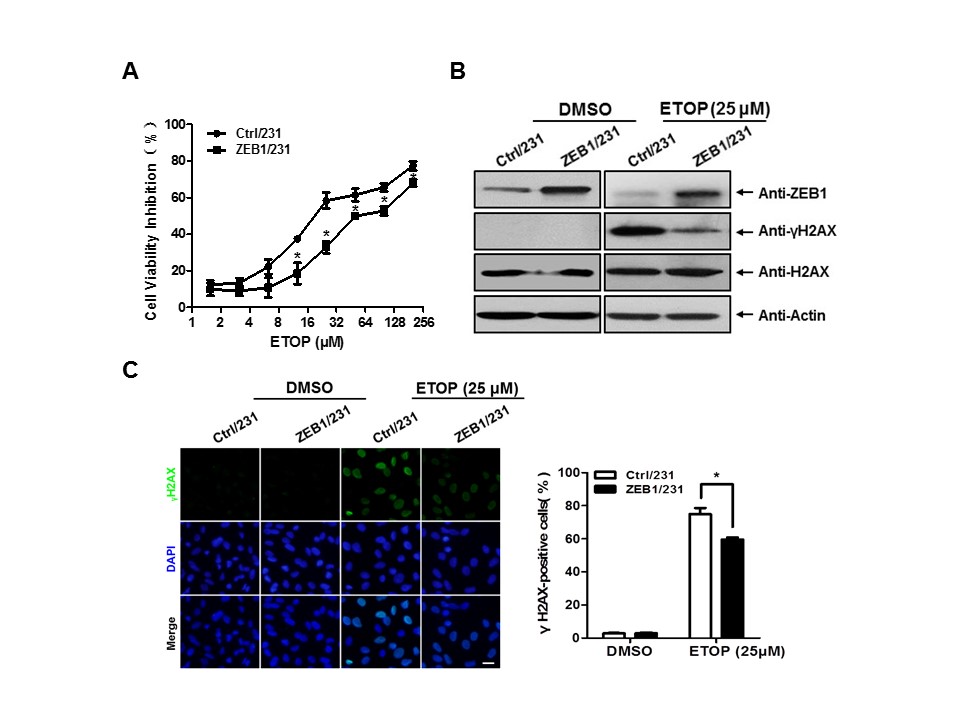


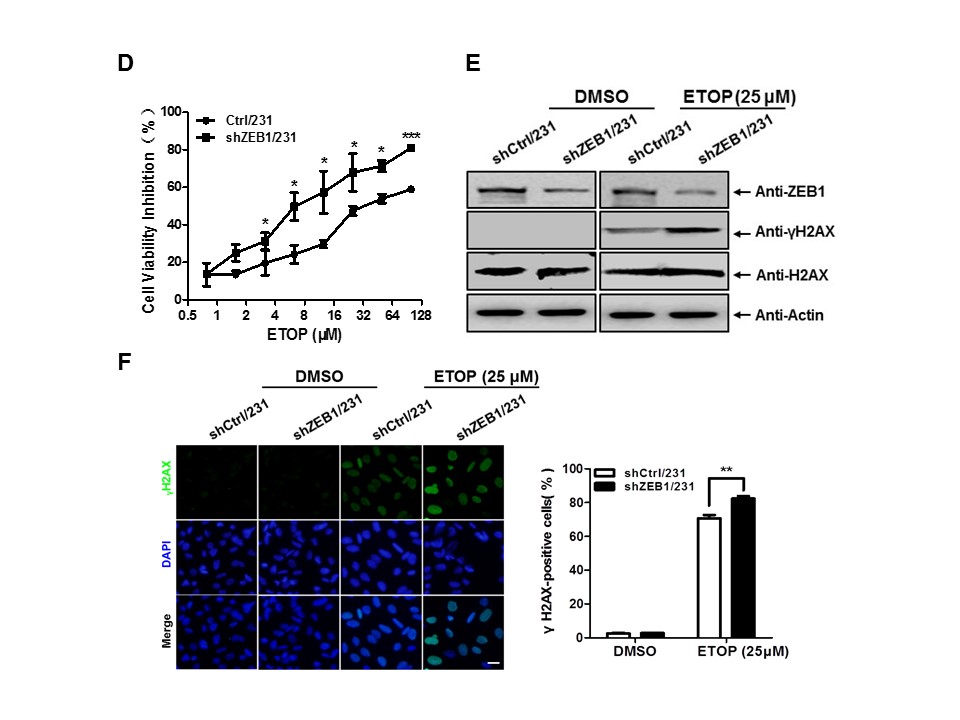


Figure S2 ZEB1 confers resistance to ETOP-induced cell viability inhibition, γH2AX levels and formation of γH2AX foci in MDA-MB-231 cells. (**a**-**c**) ZEB1/231 and Ctrl/231 cells were treated with different concentrations of ETOP for 48 h, respectively. (**a**) ETOP-induced cell viability inhibition was determined by CCK-8 assays. **P* < 0.05 vs respective control in one-way ANOVA followed by Tukey’s Honestly Significant Difference test. (**b**) The expression of γH2AX protein was determined by immunoblotting and normalized to the levels of H2AX. (**c**) ETOP-induced γH2AX nucleic foci were measured by immunofluorescent staining. At least 500 nuclei were counted and the percentage of γH2AX-positive nuclei was determined. **P* < 0.05 vs respective control in Student’s *t*-test. (**d**-**f**) shZEB1/231 and shCtrl/231 cells were treated with different concentrations of ETOP for 48 h, respectively. (**d**) ETOP-induced cell viability inhibition was determined by cell viability assay. **P* < 0.05, ****P* < 0.001 vs respective control in one-way ANOVA followed by Tukey’s Honestly Significant Difference test. (**e**) The expression of γH2AX protein was determined by immunoblotting and normalized to the levels of H2AX. (**f**) ETOP-induced γH2AX nucleic foci were measured by immunofluorescent staining. At least 500 nuclei were counted and the percentage of γH2AX-postitive nuclei was determined. ***P* < 0.01 vs respective control in Student’s *t*-test.


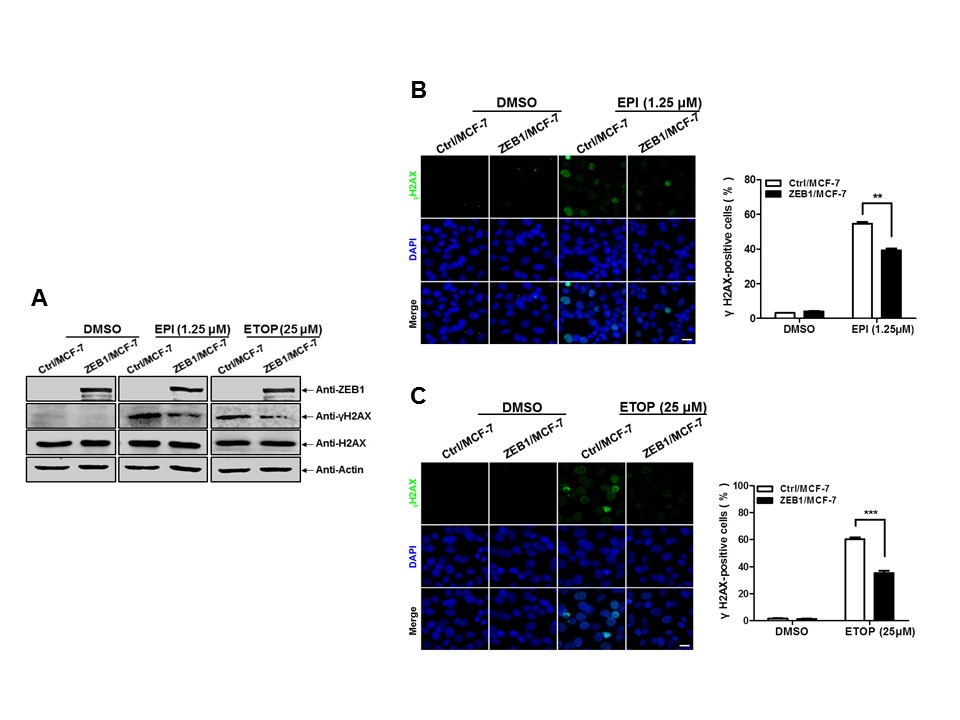


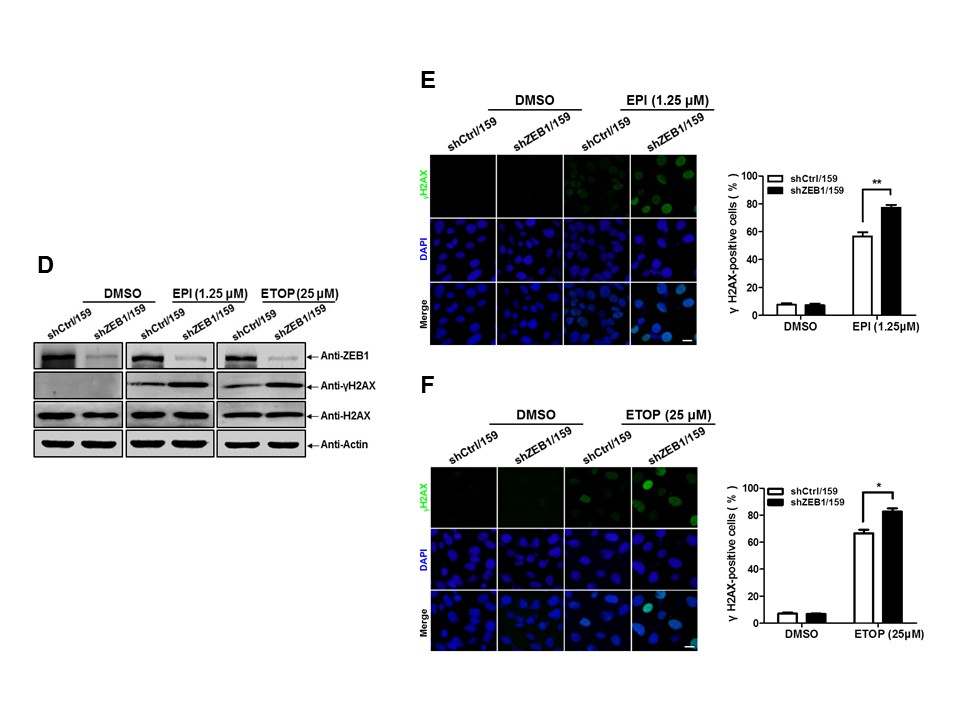


Figure S3 ZEB1 confers resistance to EPI- and ETOP-induced cell viability inhibition, γH2AX levels and formation of γH2AX foci in MCF-7 and SUM-159 cells. (**a**-**c**) ZEB1/MCF-7 and Ctrl/MCF-7 cells were treated with different concentrations of EPI or ETOP for 48 h, respectively. (**a**) EPI- or ETOP-induced expression of γH2AX protein was determined by immunoblotting and normalized to the levels of H2AX. (**b**) EPI-induced γH2AX nucleic foci were measured by immunofluorescent staining. At least 500 nuclei were counted and the percentage of γH2AX-positive nuclei was determined. **P* < 0.05 vs respective control in Student’s *t*-test. (**c**) ETOP-induced γH2AX nucleic foci were measured by immunofluorescent staining. At least 500 nuclei were counted and the percentage of γH2AX-positive nuclei was determined. ***P* < 0.01 vs respective control in Student’s *t*-test. (**d**-**f**) shZEB1/159 and shCtrl/159 cells were treated with different concentrations of EPI or ETOP for 48 h, respectively. (**d**) EPI- or ETOP-induced expression of γH2AX protein was determined by immunoblotting and normalized to the levels of H2AX. (**e**) EPI-induced γH2AX nucleic foci were measured by immunofluorescent staining. At least 500 nuclei were counted and the percentage of γH2AX-positive nuclei was determined. ***P* < 0.01 vs respective control in Student’s *t*-test. (**f**) ETOP-induced γH2AX nucleic foci were measured by immunofluorescent staining. At least 500 nuclei were counted and the percentage of γH2AX-positive nuclei was determined. ***P* < 0.01 vs respective control in Student’s *t*-test.


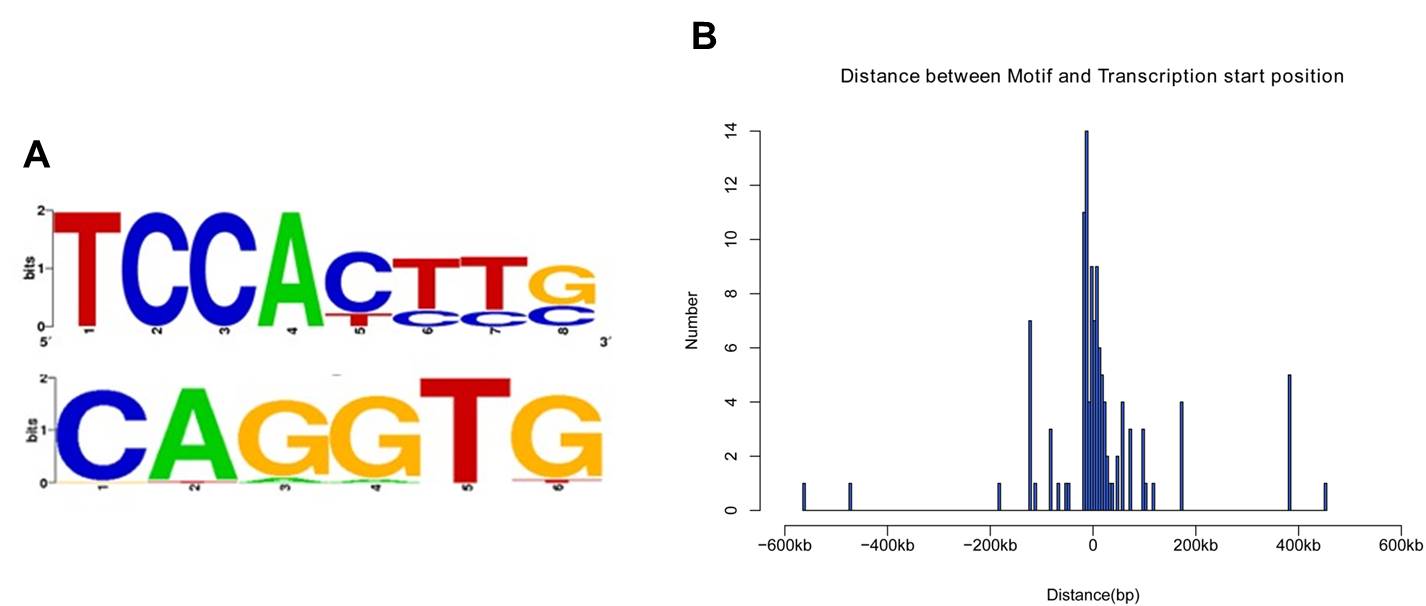


Figure S4 Distribution of ZEB1 bound promoter regions and the ZEB1 matrix site. (**a**) Weblogo of the ZEB1 matrix site identified by MEME, comprising multiple permutations of a high-affinity, E_2_-box-like consensus site. (**b**) Histogram showing distribution of the defined peak regions of ZEB1-bound promoters identified by ChIP-seq in relation to the transcriptional start site (TSS). Most of the ZEB1-bound regions and ZEB1 matrix sites contained within these defined peak regions are localized within 2 kb of the TSS.


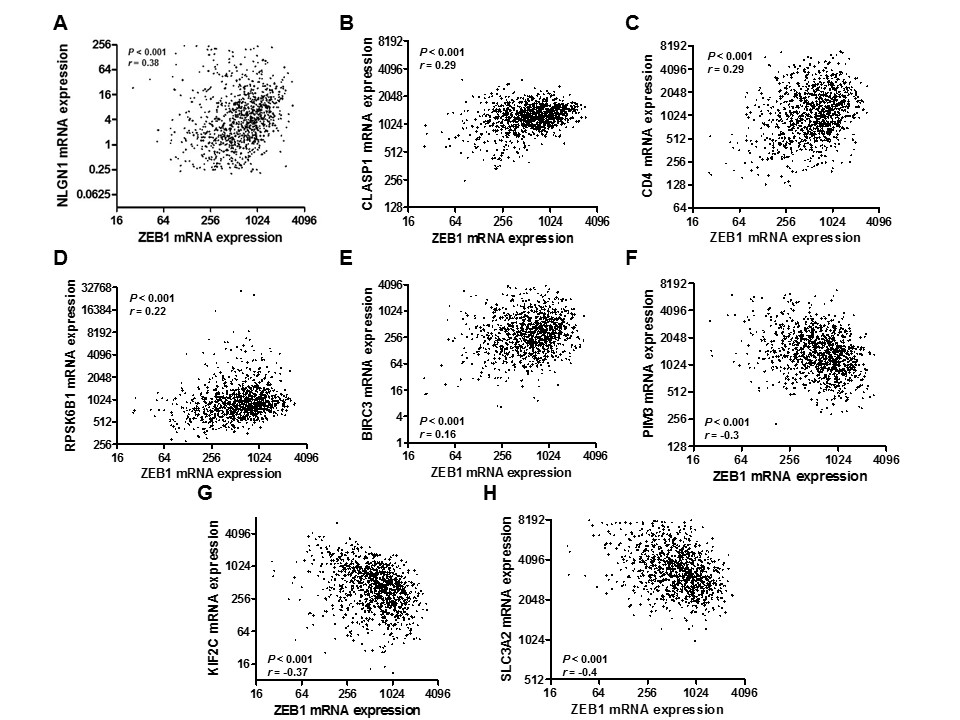


Figure S5 ZEB1 regulates chemoresistance-related genes. (**a**-**e**) TCGA database analysis indicates a positive correlation between the mRNA levels of ZEB1 and NLGN1 (**a**), CLASP1 (**b**), CD4 (**c**), RPSK6B1 (**d**), and BIRC3 (**e**). Statistical signiﬁcance was determined by Spearman rank correlation analysis. (**f**-**h**) TCGA database analysis indicates a negative correlation between the mRNA levels of ZEB1 and PIM3 (**f**), KIF2C (**g**) and SLC3A2 (**h**). Statistical signiﬁcance was determined by Spearman rank correlation analysis.


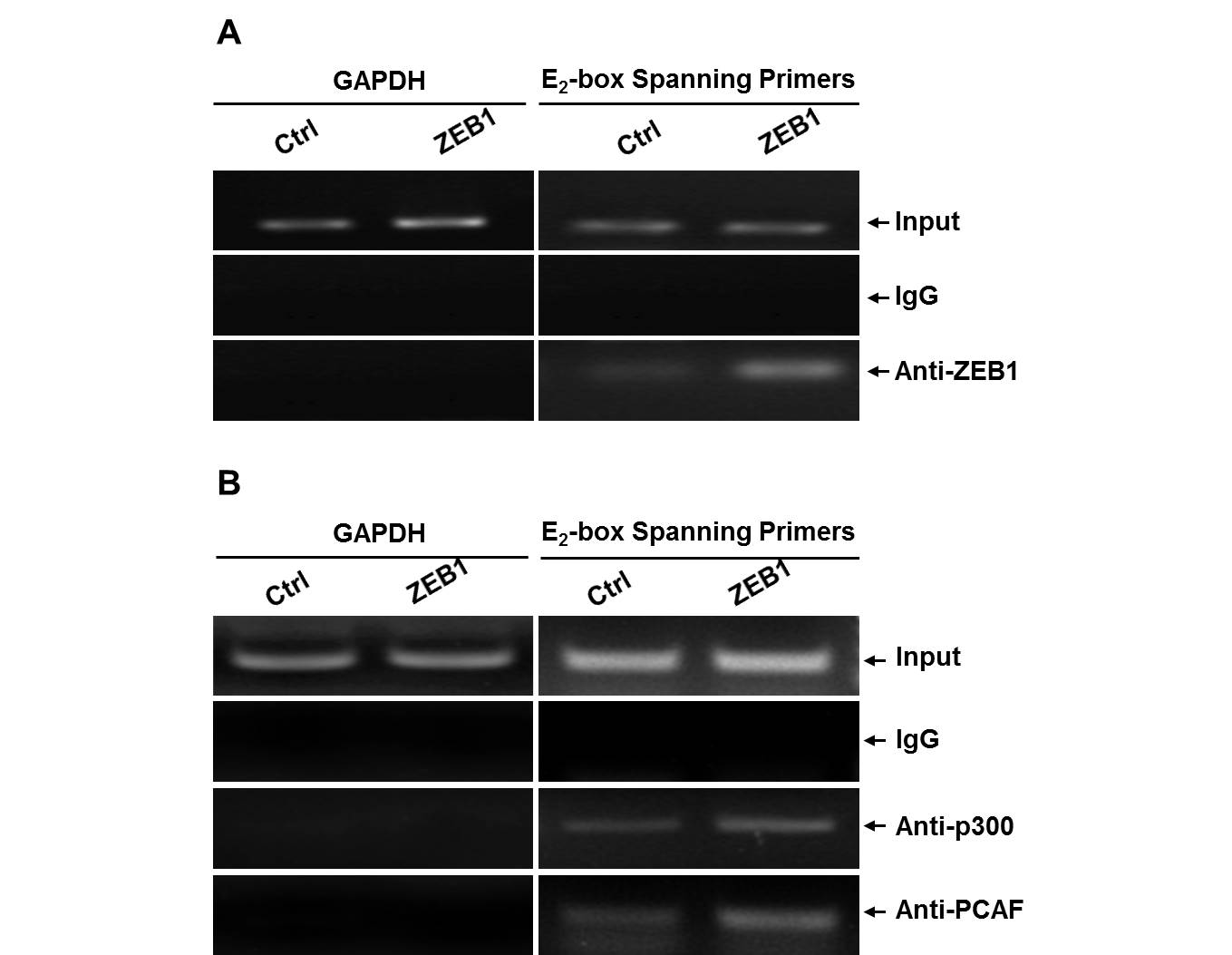


Figure S6 ZEB1 recruits to the ATM promoter in an E_2_-box dependent manner by forming a complex with p300 and PCAF. (**a**) The association of ZEB1 with the proximal ATM promoter was analyzed by ChIP assay. The amplified sequence of the ATM promoter fragment containing E_2_-box element is shown. Input DNA amounts were confirmed by equal loading of chromatin. (**b**) The association of p300 and PCAF with the proximal ATM promoter was analyzed by ChIP assay. The amplified sequence of the ATM promoter fragment containing E_2_-box element is shown. Input DNA amounts were confirmed by equal loading of chromatin.


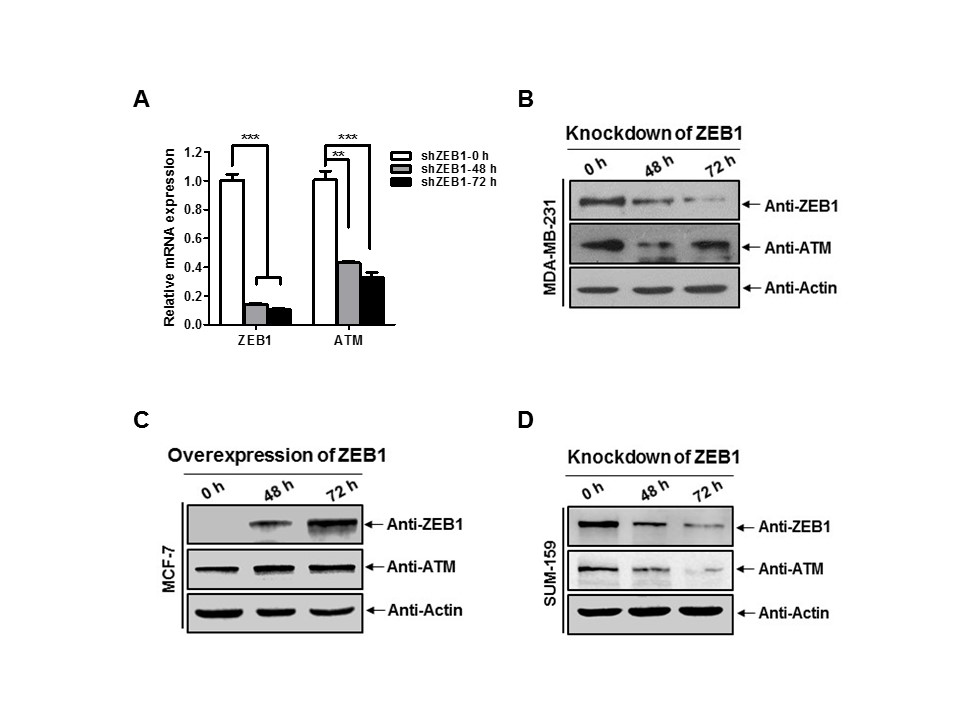


Figure S7 ZEB1 induces ATM expression at mRNA and protein levels. (**a**,**b**) MDA-MB-231 cells were transiently transfected with the specific shRNA targeting ZEB1 or scramble shRNA. At the indicated time points, expression of ZEB1 and ATM were verified by quantitative PCR (**a**) and immunoblotting (**b**) and normalized to the levels of β-actin. ***P* < 0.01, ****P* < 0.001 vs respective control in one-way ANOVA followed by Tukey’s Honestly Significant Difference test. (**c**) MCF-7 cells were transiently transfected with the ZEB1 expression plasmid or vector control. At the indicated time points, expression of ZEB1 and ATM were verified by immunoblotting and normalized to the levels of β-actin. (**d**) SUM-159 cells were transiently transfected with the specific shRNA targeting ZEB1 or scramble shRNA. At the indicated time points, expression of ZEB1 and ATM were verified by immunoblotting and normalized to the levels of β-actin.


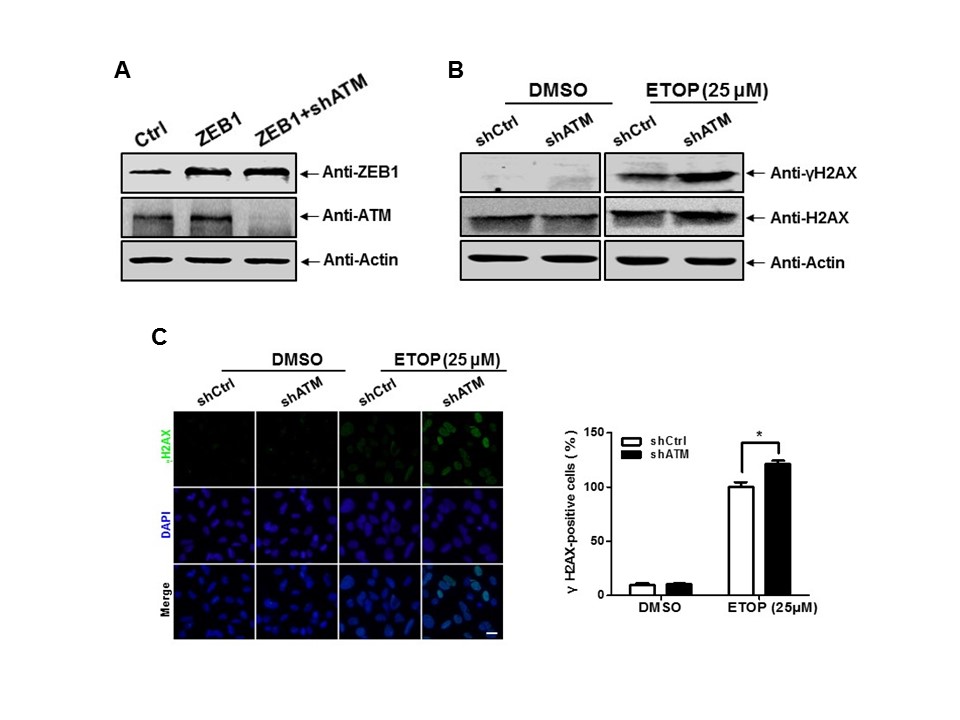


Figure S8 ATM is required for ZEB1-mediated chemoresistance. (**a-c**) The specific shRNA targeting ATM or scramble shRNA was introduced into ZEB1/231 cells, followed by treatment with different concentrations of ETOP. (**a**) The expression of ATM was verified by immunoblotting and normalized to the levels of β-actin. (**b**) ETOP-induced expression of γH2AX protein was determined by immunoblotting and normalized to the levels of H2AX. (**c**) ETOP-induced formation of γH2AX nucleic foci was measured by immunofluorescent staining. At least 500 nuclei were counted and the percentage of γH2AX-postitive nuclei was determined. **P* < 0.05 vs respective control in Student’s *t*-test.

Table S1 Summary statistics of ZEB1 ChIP-seq peaks in selected ZEB1 target genes.

| **NO.** | | **Gene Name** | | | **Gene ID** | | **Fold enrichment** | | **Peak Region** | | **Distance to TSS** | | **ZEB1 consensus site** | | |
| --- | --- | --- | --- | --- | --- | --- | --- | --- | --- | --- | --- | --- | --- | --- | --- |
|  | |  | | |  | |  | |  | |  | |  | | |
| 1 | | MIR1977 | | | NR_031741 | | 24.48 | | chr1:569728-570125 | | N/A | | N/A | | |
| 2 | | LOC26080 | | | NR_027278 | | 13.64 | | chrUn_gl000212:40354-41367 | | 828 | | CAGGTG | | |
| 3 | | SENP5 | | | NM_152699 | | 13.54 | | chr3:196625239-196626134 | | -1021 | | CAGGTG | | |
| 4 | | PRR5 | | | NM_001017528 | | 12.95 | | chr22:45126798-45127446 | | 862 | | CAGGTG | | |
| 5 | | PRR5-ARHGAP8 | | | NM_181334 | | 12.95 | | chr22:45126798-45127446 | | -388 | | CAGGTG | | |
| 6 | | PDE6B | | | NM_000283 | | 12.07 | | chr4:616322-616937 | | 911 | | CAGGTG | | |
| 7 | | ATP11A | | | NM_015205 | | 11.29 | | chr13:113369568-113370285 | | 158 | | CAGCTG | | |
| 8 | | TMEM80 | | | NM_001042463 | | 11.15 | | chr11:693915-694734 | | -204 | | CAGCTG | | |
| 9 | | DEAF1 | | | NM_021008 | | 11.15 | | chr11:693915-694734 | | -581 | | CAGGTG | | |
| 10 | | EPS8L2 | | | NM_022772 | | 11.15 | | chr11:693915-694734 | | 167 | | CAGGTG | | |
| 11 | | TMEM80 | | | NM_174940 | | 11.15 | | chr11:693915-694734 | | 559 | | CACGTG | | |
| 12 | | DGCR6 | | | NM_005675 | | 10.56 | | chr22:18879516-18880194 | | 862 | | CACCTG | | |
| 13 | | SLC3A2 | | | NM_001012661 | | 10.31 | | chr11:62608316-62609696 | | 1347 | | CAGGTG | | |
| 14 | | STX5 | | | NM_003164 | | 10.31 | | chr11:62608316-62609696 | | 150 | | CAGGTG | | |
| 15 | | WDR74 | | | NM_018093 | | 10.31 | | chr11:62608316-62609696 | | 30 | | CAGGTG | | |
| 16 | | SNORD22 | | | NR_000008 | | 10.31 | | chr11:62608316-62609696 | | -126 | | CAGGTG | | |
| 17 | | SNORD29 | | | NR_002559 | | 10.31 | | chr11:62608316-62609696 | | -376 | | CAGGTG | | |
| 18 | | SNORD31 | | | NR_002560 | | 10.31 | | chr11:62608316-62609696 | | -951 | | CAGGTG | | |
| 19 | | SNORD30 | | | NR_002561 | | 10.31 | | chr11:62608316-62609696 | | -612 | | CAGGTG | | |
| 20 | | SNORD28 | | | NR_002562 | | 10.31 | | chr11:62608316-62609696 | | -381 | | CAGGTG | | |
| **NO.** | | **Gene Name** | **Gene ID** | | **Fold enrichment** | | **Peak Region** | | **Distance to TSS** | | **ZEB1 consensus site** | | |  |  |
|  | |  |  | |  | |  | |  | |  | | |  |  |
| 21 | | SNORD27 | NR_002563 | | 10.31 | | chr11:62608316-62609696 | | 7 | | CAGGTG | |  |  |  |
| 22 | | SNORD26 | NR_002564 | | 10.31 | | chr11:62608316-62609696 | | -1545 | | CAGCTG | |  |  |  |
| 23 | | SNORD25 | NR_002565 | | 10.31 | | chr11:62608316-62609696 | | -1280 | | CAGCTG | |  |  |  |
| 24 | | SNHG1 | NR_003098 | | 10.31 | | chr11:62608316-62609696 | | 812 | | CAGGTG | |  |  |  |
| 25 | | PCBD2 | NM_032151 | | 10.21 | | chr5:134259712-134260811 | | 988 | | CAGGTG | |  |  |  |
| 26 | | MAMDC2 | NM_153267 | | 10 | | chr9:72652791-72653695 | | -544 | | CAGGTG | |  |  |  |
| 27 | | ATXN2 | NM_002973 | | 9.53 | | chr12:112049898-112050331 | | -1109 | | CAGGTG | |  |  |  |
| 28 | | DCC | NM_005215 | | 9.29 | | chr18:50318980-50319587 | | N/A | | N/A | |  |  |  |
| 29 | | DSCR3 | NM_006052 | | 9.17 | | chr21:38595883-38596437 | | -948 | | CAGGTG | |  |  |  |
| 30 | | DSCR9 | NR_026719 | | 9.17 | | chr21:38595883-38596437 | | 252 | | CAGGTG | |  |  |  |
| 31 | | ISPD | NM_001101417 | | 9.17 | | chr7:16376478-16377076 | | 65 | | CAGGTG | |  |  |  |
| 32 | | IFI6 | NM_002038 | | 8.97 | | chr1:27995245-27996226 | | 967 | | CAGGTG | |  |  |  |
| 33 | | ZNF385B | NM_001113397 | | 8.87 | | chr2:180587491-180587986 | | 1214 | | CAGGTG | |  |  |  |
| 34 | | C7orf63 | NM_001039706 | | 8.87 | | chr7:89947053-89947669 | | -379 | | CAGGTG | |  |  |  |
| 35 | | FIBCD1 | NM_001145106 | | 8.87 | | chr9:133816369-133816871 | | 224 | | CAGCTG | |  |  |  |
| 36 | | BTRC | NM_033637 | | 8.73 | | chr10:103124119-103125135 | | -458 | | CAGGTG | |  |  |  |
| 37 | | IRX3 | NM_024336 | | 8.67 | | chr16:54320102-54320573 | | 358 | | CAGGTG | |  |  |  |
| 38 | | PIM3 | NM_001001852 | | 8.67 | | chr22:50364008-50364825 | | -401 | | CAGGTG | |  |  |  |
| 39 | | CLASP1 | NM_001142273 | | 8.67 | | chr2:122288282-122289026 | | 2560 | | CAGGTG | |  |  |  |
| 40 | | RNU4ATAC | NR_023343 | | 8.67 | | chr2:122288282-122289026 | | -853 | | CAGCTG | |  |  |  |

| **NO.** | **Gene Name** | **Gene ID** | **Fold enrichment** | **Peak Region** | **Distance to TSS** | **ZEB1 consensus site** |
| --- | --- | --- | --- | --- | --- | --- |
|  |  |  |  |  |  |  |
| 41 | NCRNA00152 | NR_024204 | 8.67 | chr2:87776102-87776616 | -811 | CAGCTG |
| 42 | C3orf21 | NM_152531 | 8.67 | chr3:194934756-194935380 | -152 | CAGGTG |
| 43 | TBCK | NM_001163435 | 8.67 | chr4:107194616-107195159 | -557 | CAGCTG |
| 44 | PTPN12 | NM_001131008 | 8.67 | chr7:77161205-77161803 | -1865 | CAGGTG |
| 45 | ARHGEF10 | NM_014629 | 8.67 | chr8:1805930-1806688 | -498 | CAGGTG |
| 46 | GRHL3 | NM_021180 | 8.64 | chr1:24671285-24672131 | 9 | CAGCTG |
| 47 | C1orf201 | NM_178122 | 8.64 | chr1:24671285-24672131 | 1895 | CAGGTG |
| 48 | ABLIM2 | NM_001130083 | 8.62 | chr4:8035706-8036468 | 183 | CAGCTG |
| 49 | PRKAG2 | NM_001040633 | 8.59 | chr7:151366756-151367339 | 368 | CAGGTG |
| 50 | SMYD3 | NM_001167740 | 8.57 | chr1:246106980-246107528 | -1241 | CAGGTG |
| 51 | C17orf105 | NM_001136483 | 8.33 | chr17:41867704-41868159 | -1021 | CAGGTG |
| 52 | DUSP3 | NM_004090 | 8.33 | chr17:41867704-41868159 | 1103 | CAGGTG |
| 53 | MPP3 | NR_003562 | 8.33 | chr17:41867704-41868159 | 1070 | CAGGTG |
| 54 | C1orf159 | NM_017891 | 8.33 | chr1:1058630-1059769 | -220 | CAGGTG |
| 55 | LOC732275 | NR_024406 | 8.16 | chr16:86371479-86372358 | 317 | CAGCTG |
| 56 | CD4 | NM_000616 | 8.11 | chr12:6944779-6945395 | -569 | CAGGTG |
| 57 | GNB3 | NM_002075 | 8.11 | chr12:6944779-6945395 | 85 | CAGGTG |
| 58 | USP5 | NM_003481 | 8.11 | chr12:6944779-6945395 | 1464 | CAGGTG |
| 59 | LEPREL2 | NM_014262 | 8.11 | chr12:6944779-6945395 | 564 | CAGGTG |
| 60 | GPR162 | NM_014449 | 8.11 | chr12:6944779-6945395 | -373 | CAGCTG |

| **NO.** | **Gene Name** | **Gene ID** | **Fold enrichment** | **Peak Region** | **Distance to TSS** | **ZEB1 consensus site** |
| --- | --- | --- | --- | --- | --- | --- |
|  |  |  |  |  |  |  |
| 61 | CDCA3 | NM_031299 | 8.11 | chr12:6944779-6945395 | N/A | N/A |
| 62 | EXD3 | NM_017820 | 8 | chr9:140222239-140223047 | -446 | CAGGTG |
| 63 | STK10 | NM_005990 | 7.86 | chr5:171566145-171566667 | -156 | CAGGTG |
| 64 | GTDC1 | NM_001164629 | 7.81 | chr2:144960715-144961133 | 821 | CAGGTG |
| 65 | KCNJ4 | NM_004981 | 7.8 | chr22:38808490-38809151 | -534 | CAGGTG |
| 66 | LOC400927 | NR_002821 | 7.8 | chr22:38808490-38809151 | 317 | CAGGTG |
| 67 | FRYL | NM_015030 | 7.8 | chr4:48662327-48663041 | 239 | CAGGTG |
| 68 | PTPRQ | NM_001145026 | 7.76 | chr12:80976568-80977116 | 1534 | CAGCTG |
| 69 | ENPP3 | NM_005021 | 7.74 | chr6:131960513-131961120 | -256 | CAGCTG |
| 70 | MED23 | NM_015979 | 7.74 | chr6:131960513-131961120 | -1158 | CAGGTG |
| 71 | FBXL4 | NM_012160 | 7.74 | chr6:99313931-99314471 | -1034 | CAGGTG |
| 72 | FLJ36000 | NR_027084 | 7.69 | chr17:21904423-21905088 | 1314 | CAGGTG |
| 73 | CTAGE5 | NM_005930 | 7.5 | chr14:39822222-39822716 | 1245 | CAGGTG |
| 74 | PTPRK | NM_001135648 | 7.5 | chr6:128587624-128588150 | 2 | CAGGTG |
| 75 | TNKS | NM_003747 | 7.5 | chr8:9551317-9551783 | -1412 | CAGGTG |
| 76 | ARL4D | NM_001661 | 7.43 | chr17:41465738-41466671 | -944 | CAGGTG |
| 77 | LOC100130581 | NR_027412 | 7.43 | chr17:41465738-41466671 | 191 | CAGGTG |
| 78 | PDE4DIP | NM_001002810 | 7.35 | chr1:144966104-144966686 | 142 | CAGCTG |
| 79 | DBNDD1 | NM_001042610 | 7.26 | chr16:90098126-90099594 | -346 | CAGGTG |
| 80 | C16orf3 | NM_001214 | 7.26 | chr16:90098126-90099594 | -133 | CAGGTG |

| **NO.** | **Gene Name** | **Gene ID** | **Fold enrichment** | **Peak Region** | **Distance to TSS** | **ZEB1 consensus site** |
| --- | --- | --- | --- | --- | --- | --- |
|  |  |  |  |  |  |  |
| 81 | GAS8 | NM_001481 | 7.26 | chr16:90098126-90099594 | -69 | CAGGTG |
| 82 | LOC100130015 | NR_027335 | 7.26 | chr16:90098126-90099594 | -101 | CAGGTG |
| 83 | NBR1 | NM_005899 | 7.23 | chr17:41381406-41382570 | 842 | CAGCTG |
| 84 | TMEM106A | NM_145041 | 7.23 | chr17:41381406-41382570 | -720 | CAGCTG |
| 85 | PHF2 | NM_005392 | 7.14 | chr9:96434487-96435257 | 243 | CAGGTG |
| 86 | TRIP6 | NM_003302 | 7.08 | chr7:100449635-100450531 | -504 | CAGGTG |
| 87 | SLC12A9 | NM_020246 | 7.08 | chr7:100449635-100450531 | 28 | CAGGTG |
| 88 | RPL31 | NM_001098577 | 7.05 | chr2:101655087-101655727 | 755 | CAGCTG |
| 89 | TBC1D8 | NM_001102426 | 7.05 | chr2:101655087-101655727 | -1216 | CAGGTG |
| 90 | PADI2 | NM_007365 | 7.03 | chr1:17448326-17448952 | 264 | CAGGTG |
| 91 | C20orf160 | NM_080625 | 7.03 | chr20:30607846-30608496 | -714 | CAGGTG |
| 92 | BIRC3 | NM_001165 | 6.98 | chr11:102191447-102191786 | 133 | CAGGTG |
| 93 | RPS6KB1 | NM_003161 | 6.98 | chr17:58045197-58045894 | 469 | CAGGTG |
| 94 | RNFT1 | NM_016125 | 6.98 | chr17:58045197-58045894 | -282 | CAGGTG |
| 95 | DHX40P1 | NR_002924 | 6.98 | chr17:58045197-58045894 | 565 | CAGGTG |
| 96 | ATM | NM_000051 | 6.88 | chr11:108108119-108108633 | -251 | CAGGTG |
| 97 | NPAT | NM_002519 | 6.88 | chr11:108108119-108108633 | -1614 | CACCTG |
| 98 | OR52K2 | NM_001005172 | 6.82 | chr11:4455607-4456317 | 115 | CACCTG |
| 99 | ZBTB46 | NM_025224 | 6.82 | chr20:62446349-62447802 | -358 | CAGCTG |
| 100 | GLYATL1 | NM_080661 | 6.67 | chr11:58712783-58713403 | -223 | CAGGTG |

| **NO.** | **Gene Name** | **Gene ID** | **Fold enrichment** | **Peak Region** | **Distance to TSS** | **ZEB1 consensus site** |
| --- | --- | --- | --- | --- | --- | --- |
|  |  |  |  |  |  |  |
| 101 | MLL3 | NM_170606 | 6.67 | chr7:152084956-152085569 | -542 | CAGGTG |
| 102 | CLIP1 | NM_198240 | 6.62 | chr12:122920363-122920841 | N/A | N/A |
| 103 | NALCN | NM_052867 | 6.62 | chr13:101924377-101924851 | -49 | CAGGTG |
| 104 | TAF12 | NM_001135218 | 6.6 | chr1:28974772-28975863 | -250 | CAGGTG |
| 105 | GMEB1 | NM_006582 | 6.6 | chr1:28974772-28975863 | 1114 | CAGGTG |
| 106 | RNU11 | NR_004407 | 6.6 | chr1:28974772-28975863 | 105 | CAGCTG |
| 107 | LOC283788 | NR_027436 | 6.5 | chrUn_gl000219:59009-59631 | 1423 | CACCTG |
| 108 | ARHGEF10L | NM_001011722 | 6.43 | chr1:18001702-18003055 | 402 | CAGGTG |
| 109 | C11orf49 | NM_001003676 | 6.25 | chr11:47129326-47130007 | 789 | CAGGTG |
| 110 | FGF14 | NM_175929 | 6.25 | chr13:102901569-102902144 | -145 | CAGCTG |
| 111 | NBPF14 | NM_015383 | 6.25 | chr1:148008958-148010219 | 348 | CAGGTG |
| 112 | PCDH7 | NM_001173523 | 6.25 | chr4:30819553-30820164 | 102 | CAGGTG |
| 113 | ZCCHC7 | NM_032226 | 6.2 | chr9:37224505-37225278 | 287 | CAGGTG |
| 114 | ARHGEF18 | NM_001130955 | 6.1 | chr19:7515153-7516077 | 943 | CAGGTG |
| 115 | PTPRN2 | NM_002847 | 6.06 | chr7:157940035-157940753 | N/A | N/A |
| 116 | KLF12 | NM_007249 | 6.05 | chr13:74310957-74311471 | -26 | CACCTG |
| 117 | ATP4B | NM_000705 | 5.97 | chr13:114319656-114321124 | 188 | CAGGTG |
| 118 | GRK1 | NM_002929 | 5.97 | chr13:114319656-114321124 | 949 | CACGTG |
| 119 | NLGN1 | NM_014932 | 5.92 | chr3:173601748-173602302 | -184 | CAGGTG |
| 120 | THNSL1 | NM_024838 | 5.83 | chr10:25298832-25299439 | 0 | CACGTG |

| **NO.** | **Gene Name** | **Gene ID** | **Fold enrichment** | **Peak Region** | **Distance to TSS** | **ZEB1 consensus site** |
| --- | --- | --- | --- | --- | --- | --- |
|  |  |  |  |  |  |  |
| 121 | ENKUR | NM_145010 | 5.83 | chr10:25298832-25299439 | 1305 | CAGGTG |
| 122 | LPCAT1 | NM_024830 | 5.77 | chr5:1524612-1525153 | 19 | CAGGTG |
| 123 | PLDN | NM_012388 | 5.73 | chr15:45896901-45897431 | 213 | CAGCTG |
| 124 | LOC100130581 | NR_027412 | 5.65 | chr17:41437984-41439046 | 191 | CAGGTG |
| 125 | MAP3K13 | NM_004721 | 5.62 | chr3:185196738-185197323 | -110 | CAGGTG |
| 126 | TMEM41A | NM_080652 | 5.62 | chr3:185196738-185197323 | 1259 | CAGGTG |
| 127 | ROCK1 | NM_005406 | 5.6 | chr18:18510800-18513264 | -879 | CAGGTG |
| 128 | DTWD2 | NM_173666 | 5.36 | chr5:118330372-118331306 | -1161 | CAGGTG |
| 129 | ZNF414 | NM_001146175 | 5.32 | chr19:8594376-8595156 | -961 | CAGCTG |
| 130 | MYO1F | NM_012335 | 5.32 | chr19:8594376-8595156 | -779 | CAGGTG |
| 131 | ZNF414 | NM_032370 | 5.32 | chr19:8594376-8595156 | -961 | CAGCTG |
| 132 | C7orf41 | NM_152793 | 5.26 | chr7:30197377-30198175 | -752 | CAGGTG |
| 133 | TMEM214 | NM_001083590 | 5.17 | chr2:27273509-27274393 | 582 | CAGGTG |
| 134 | OST4 | NM_001134693 | 5.17 | chr2:27273509-27274393 | -926 | CACCTG |
| 135 | AGBL5 | NM_021831 | 5.17 | chr2:27273509-27274393 | 200 | CAGCTG |
| 136 | HEATR7A | NM_001099280 | 5 | chr8:145277092-145278223 | -438 | CAGCTG |
| 137 | TEX14 | NM_031272 | 4.76 | chr17:56708626-56709670 | -1023 | CAGGTG |
| 138 | C1orf228 | NM_001145636 | 4.74 | chr1:45186733-45187724 | -432 | CAGCTG |
| 139 | KIF2C | NM_006845 | 4.74 | chr1:45186733-45187724 | -507 | CAGGTG |
| 140 | KANK2 | NM_001136191 | 4.57 | chr19:11319738-11322092 | 17 | CAGGTG |

| **NO.** | **Gene Name** | **Gene ID** | **Fold enrichment** | **Peak Region** | **Distance to TSS** | **ZEB1 consensus site** |
| --- | --- | --- | --- | --- | --- | --- |
|  |  |  |  |  |  |  |
| 141 | DOCK6 | NM_020812 | 4.57 | chr19:11319738-11322092 | -1222 | CAGCTG |
| 142 | RBMS3 | NM_001003792 | 4.55 | chr3:29706979-29707795 | 1732 | CAGGTG |
| 143 | MIR596 | NR_030326 | 4.55 | chr8:1773098-1774192 | -31 | CAGGTG |
| 144 | GPR55 | NM_005683 | 4.44 | chr2:231770424-231771194 | -139 | CAGGTG |
| 145 | DHX32 | NM_018180 | 4.05 | chr10:127582173-127583574 | -4 | CAGGTG |
| 146 | FANK1 | NM_145235 | 4.05 | chr10:127582173-127583574 | -95 | CAGGTG |
| 147 | GLIS1 | NM_147193 | 3.31 | chr1:54021262-54022909 | 475 | CAGGTG |

Table S2 ZEB1 expression and clinicopathologic features of breast tumors.

| **Clinicopathologic features** | **Number (n=233)** | **ZEB1 expression** | | ***P* value** |
| --- | --- | --- | --- | --- |
|  |  | **High** | **Low** |  |
| **Age (years)** |  |  |  | *P* = 0.407 |
| ≤40 | 93 | 46 | 47 |  |
| ＞40 | 140 | 77 | 63 |  |
| **Tumor size (cm)** |  |  |  | *P* = 0.057 |
| ≤2.0 | 19 | 10 | 9 |  |
| ＞2.0, ≤5.0 | 96 | 42 | 54 |  |
| ＞5.0 | 118 | 71 | 47 |  |
| **Lymph node metastasis** |  |  |  | *P* = 0.032 |
| Positive | 110 | 68 | 42 |  |
| Negative | 94 | 44 | 50 |  |
| Unknown | 29 | 11 | 18 |  |
| **ER status** |  |  |  | *P* = 0.048 |
| Positive | 114 | 51 | 63 |  |
| Negative | 105 | 61 | 44 |  |
| Unknown | 14 | 11 | 3 |  |
| **PR status** |  |  |  | *P* = 0.481 |
| Positive | 95 | 46 | 49 |  |
| Negative | 124 | 66 | 58 |  |
| Unknown | 14 | 11 | 3 |  |
| **HER2 status** |  |  |  | *P* = 0.186 |
| Positive | 128 | 71 | 57 |  |
| Negative | 66 | 30 | 36 |  |
| Unknown | 39 | 22 | 17 |  |
